# Supplementary figures and images for: The never-ending patient journey of chronically ill patients: A qualitative case study on touchpoints in relation to patient-centered care
Source: PLoS One. 2023 May 17;18(5):e0285872. doi: 10.1371/journal.pone.0285872 (PMC10191281; doi:10.1371/journal.pone.0285872)

**S1 Fig. Visualization of the baseline patient journey.**

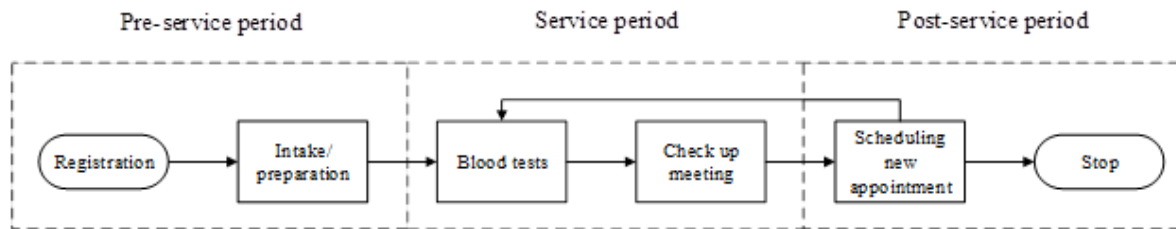

Supplement: S1 Fig — (PDF) [file pone.0285872.s001.pdf]
