# Supplementary material for: The never-ending patient journey of chronically ill patients: A qualitative case study on touchpoints in relation to patient-centered care
Source: PLoS One. 2023 May 17;18(5):e0285872. doi: 10.1371/journal.pone.0285872 (PMC10191281; doi:10.1371/journal.pone.0285872)
Supplement: S1 File — (DOCX) [file pone.0285872.s002.docx]

**S1 File. A 32-item checklist for reporting qualitative studies (COREQ).**

| **Item** | **Description** | **Check** |
| --- | --- | --- |
| **Domain 1: Research team and reflexivity** | | |
| *Personal characteristics* | | |
| 1. Interviewer | Which author/s conducted the interviews and/or observations? | Author 2 |
| 2. Credentials | What were the researcher’s credentials? | Author 1: MSc  Author 2: MSc  Author 3: Dr.  Author 4: Prof. dr. ir.  Author 5: MSc |
| 3. Occupation | What was the occupation at the time of the study? | Author 1: Junior researcher  Author 2: Master’s student  Author 3: Research coordinator  Author 4: Endowed professor  Author 5: Department manager |
| 4. Gender | Was the researcher male or female? | Author 1: Female  Author 2: Male  Author 3: Male  Author 4: Male  Author 5: Female |
| 5. Experience and training | What experience or training did the researcher have? | Courses on Qualitative Research Methods at Tilburg University and  previous experience during thesis writing |
| *Relationship with participants* | | |
| 6. Relationship established | Was a relationship established prior to study commencement? | A professional relationship was established with internists of the department of Internal Medicine treating chronically ill patients |
| 7. Participant knowledge of the researcher | What did the participants know about the researcher? | The personal interest of the researchers and purpose of the study was explained before the data collection started |
| 8. Researcher characteristics | What characteristics were reported about the researcher? | Interest in research topic, occupation, reason for research |
| **Domain 2: Study design** | | |
| *Theoretical framework* | | |
| 9. Methodological orientation and Theory | What methodological orientation was stated to underpin the study? | Thematic analysis |
| *Participant selection* | | |
| 10. Sampling | How were participants selected? | Convenience and Purposive sampling |
| 11. Method of approach | How were participants approached? | Telephone, face-to-face, e-mail |
| 12. Sample size | How many participants were in the study? | 8 |
| 13. Non-participation | How many people refused to participate or dropped out? Reasons? | 4 (Reasons for not participating that were given were: no interest in participation or having not sufficient time for the interview). |
| *Setting* | | |
| 14. Setting of data collection | Where was the data collected? | Workplace (hospital) and Digital (via Zoom) |
| 15. Presence of non-participants | Was anyone else present besides the participants and researchers? | No |
| 16. Description of sample | What are the important characteristics of the sample? | Gender, age, type of illness, digital literacy, Number of years living with chronic illness |
| *Data collection* | | |
| 17. Interview guide | Were questions, prompts, guides provided by the authors?  Was it pilot tested? | The topic list is added to the manuscript; the topic list was pilot tested and discussed within the study team |
| 18. Repeat interviews | Were repeat interviews carried out? If yes, how many? | No repeat interviews were carried out |
| 19. Audio recording | Did the research use audio recording to collect the data? | Yes |
| 20. Field notes | Were field notes made during and/or after the interview or observation? | Yes |
| 21. Duration | What was the duration of the interviews or observation? | Interviews: 20-55 minutes |
| 22. Data saturation | Was data saturation discussed? | Data saturation was discussed within the research team |
| 23. Transcripts returned | Were transcripts returned to participants for comment and/or correction? | Transcripts were returned to participants. We received no comments and corrections. |
| **Domain 3: Analysis and findings** | | |
| *Data analysis* | | |
| 24. Number of data coders | How many data coders coded the data? | Two (Author 2 and Author 3) |
| 25. Description of the coding list | Did authors provide a description of the coding list? | The coding list is described in the Methods section and added to the manuscript as appendix. |
| 26. Derivation of themes | Were themes identified in advance or derived from the data? | Themes were identified in advance, but we also made use of a code called ‘other’ in which relevant other themes were categorized |
| 27. Software | What software, if applicable, was used to manage the data? | Microsoft Word and Microsoft Excel |
| 28. Participant checking | Did participants provide feedback on the findings? | We asked the participants to reflect on the findings of the study and received no comments or corrections |
| *Reporting* | | |
| 29. Quotations presented | Were participant quotations presented to illustrate the themes/findings? Was each quotation identified? | We used various quotations from our participants. Moreover, we created a figure to illustrate our results. |
| 30. Data and findings consistent | Was there consistency between the data presented and the findings? | We present an analytic story where we highlight the key themes of the study |
| 31. Clarity of major themes | Were major themes clearly presented in the findings? | We present major themes in the results section |
| 32. Clarity of minor themes | Is there a description of diverse cases or discussion of minor themes? | The minor themes are supportive of the major themes and presented in the results section of the study |
