# Supplementary material for: The never-ending patient journey of chronically ill patients: A qualitative case study on touchpoints in relation to patient-centered care
Source: PLoS One. 2023 May 17;18(5):e0285872. doi: 10.1371/journal.pone.0285872 (PMC10191281; doi:10.1371/journal.pone.0285872)
Supplement: Attachment — Submitted filename: The never-ending patient journey of chronically ill patients_ A qualitative case study.pdf [file pone.0285872.s003.pdf]

The never-ending patient journey of chronically ill patients: A qualitative case study

by Review Paper

Submission date: 03-Oct-2022 12:45PM (UTC+0500)
Submission ID: 1915239428
File name: PONE-D-22-21246.docx (64.84K)
Word count: 8309
Character count: 46406

The never-ending patient journey of chronically ill patients: A qualitative case study

Abstract

Background

Healthcare professionals caring for chronically ill patients increasingly want to provide patient-centered care (PCC). By understanding each individual patient journey, they can significantly improve the quality of PCC. A patient journey consists of patient interactions, so-called touchpoints, with healthcare professionals distributed over three periods: preservice, service, and post-service period. The aim of this study was to explore which digital alternatives patients would like to see implemented into their patient journey to help healthcare professionals providing PCC.

Methods

Eight semi-structured interviews were conducted either face-to-face or via Zoom. Participants were considered if they visited the department of internal medicine and were treated for either diabetes, kidney failure, arteriosclerosis, or HIV. The interviews were analyzed utilizing the three-steps method of Miles and Huberman.

Results

The results suggest that the patient journey of chronically ill patients is a continuous cycle. Furthermore, the results showed that chronically ill patients would like to see digital alternatives for touchpoints implemented into their patient journey. These digital alternatives consisted of video calls, digitally checking in before a physical appointment, digitally selfmonitoring one's medical condition and personally uploading monitoring results into the

patient portal, and viewing their own medical status in a digital manner. Particularly, patients who were familiar with their healthcare professional(s) and were in a stable condition mostly opted for digital alternatives.

Conclusion

In the cyclical patient journey, digitalization can help put the needs and wishes of chronically ill patients at the center of care provision. It is recommended that healthcare professionals implement digital alternatives for touchpoints. Most chronically ill patients consider digital alternatives to realize to more efficient interactions with their healthcare professionals. Furthermore, digital alternatives support patients to be better informed about the progress of their chronical illness.

Background

Healthcare professionals caring for chronically ill patients increasingly want to provide patient-centered care (PCC), given that patients are demanding to have more influence and control on the treatment they receive. PCC has been a focus in healthcare research for a significant amount of time to make care more focused on the patient and thereby improve safety, efficacy, public health, and patient satisfaction [1]. PCC focuses on the patient's healthcare needs and has the goal to empower patients to become active participants in their care [1]. PCC could possibly replace the current physician-centered system with one that revolves around the patient [2]. Each patient is unique and requires treatments that suits their situation in a patient-centered way. This implies that due to continuously changing patient needs and wishes, healthcare professionals are required to innovate and change the way they deliver care regularly. Also from a societal perspective, it is important that healthcare services become more responsive to the complex needs and requirements of chronically ill patients [3]. Additionally, hospitals should keep up with the ever-increasing amount of diagnostic and

treatment options, specifically including digital opportunities. Gerteis et al. [4] introduced seven dimensions that affect PCC: 1) Respect for patients' values, preferences, and expressed needs, 2) Coordination of care, 3) Communication between patient and professionals, 4) Physical care, 5) Emotional support, 6) Involvement of family and friends, and 7) Transition and continuity of care. These dimensions provide insights in how healthcare professionals can become more involved in patient-centered quality efforts, and how patient-centered quality can be integrated into health care policy, standards, and regulations. By bringing the patient's perspective to the design and delivery of health services, professionals can improve their ability to meet patient's needs and enhance the quality of care [4].

Hospitals can also significantly improve the quality of the service provided by exploring and understanding the individual patient journey [5]. The patient journey principle stems from the customer journey principle, an often studied topic within service and marketing research [6]. The customer journey is defined as "the customer's personal interpretation of the service process and their interaction and involvement with it during their journey or flow through a series of touchpoints" [6]. In the context of healthcare, the patient journey represents patient interactions, i.e., touchpoints, with multiple healthcare professionals distributed over space and time [7].

Touchpoints are present in every patient journey and are widely discussed in service research [6]. A touchpoint is considered as a moment of contact between a firm or service provider and a customer at distinct points in the customer journey [8]. Touchpoints differ from each other in the way the contact between the patient and the healthcare professional is established. In this study, the focus will be on two categories of touchpoints. The first category of touchpoints is described as healthcare encounters [9]. These encounters could be hospital admissions, urgent care, and primary care professionals visits. For these types of touchpoints, the patient is usually required to visit the healthcare institution or healthcare

professional. Some of these healthcare encounters that are currently taking place physically at the hospital could also be performed by the patient itself. For example, the self-monitoring of blood glucose for diabetes patients which has been around for years now has positively impacted the self-management of this illness [10].

The second category of touchpoints are described as device touchpoints and are considered technological solutions that are utilized by patients and healthcare professionals [9]. These touchpoints involve, for example, online meetings with healthcare professionals, administrative tasks such as planning appointments, and informational actions like reviewing one's medical records. This category of touchpoints provide tremendous opportunities to improve patient experiences and possibly also improve the efficiency of care [11]. For example, the use of video calls was found to have the potential to improve the patient experience especially if these patients otherwise must travel long distances [12].

The patient journey can be divided into three specific periods: pre-service, service, and post-service [13]. A basic patient journey of chronically ill patients usually begins during the pre-service period with the registration of the patient at the outpatient clinic. Following, an intake consult takes place between the patient and the healthcare professional to establish a first relationship and discuss the expectations for the treatment. In numerous hospitals the physical check-in at the desk before an appointment has been replaced by digital registration screens, which is found to be more efficient and less expensive compared to having several staff members at the desk. Furthermore, it improves the general patient satisfaction [14].

In the service period of the patient journey, the focus is on the regular checkupmeetings patients with chronic illnesses tend to have. Typically, chronically ill patients have a low chance of being cured. Therefore, the main meetings they have are with their healthcare professional to check their health status and to discuss the results of previous blood tests. However, extensive physical examination is usually not needed. Due to the

Covid-19 pandemic physical meetings with healthcare professionals have increasingly been rescheduled to an online format. Ramaswamy et al. [15] showed that video appointments had been increased by 8729% during the pandemic compared to the year before. They also found that patient satisfaction with video appointments is very high and is not a barrier to shift from physical visits at the outpatient clinic to online video visits. In addition, research shows that most patients have the required knowledge and technology to participate in online checkup meetings [16].

The post-service period of the patient journey is dominated by administrative actions. These involve the planning of new appointments and other administrative tasks, as well as reviewing one's medical records. A study on patient access to medical records has found modest benefits and showed moderate improvements in doctor-patient communication, adherence, patient empowerment, and patient education [17]. Though, it was also found that some patients experienced slight confusion while reading their own records and could not understand everything that was in their file. Currently various applications of digital solutions in hospital care have been integrated into so-called online patient portals. Research has indicated that the use of these portals can lead to improvements in clinical outcomes, patient behavior, and experiences [18].

Previous analysis of the patient journey has revealed three important needs for PCC, especially for patients with chronic conditions [19]. First, the patient and the healthcare professional should develop a visible health goal. Second, they should establish clear and transparent shared decision-making. Third, they should have a closed-loop communication process [19]. More factors, for example the implementation of digital innovations, can be identified in order to create a better setting for PCC [20]. Digital developments can help improve healthcare service by improving connectivity between the patient and the healthcare

professional [21]. Currently, little is known about the perspective of chronically ill patients on the implementation of digital opportunities of their patient journey to improve the delivery of PCC.

The main aim of the present study is to ascertain the chronically ill patients' needs for digital alternatives for touchpoints between them and their healthcare professionals. Specifically, we aim to explore which digital alternatives the patients would like to see implemented into their patient journey to help healthcare professionals providing PCC. The potential digital alternatives for the touchpoints are allocated to the three periods of the patient journey: pre-service, service, and post-service period. Each period has its own characteristic touchpoints and potential digital alternatives. The digital alternatives that chronically ill patients like to see implemented can lead to higher levels of patient satisfaction as it responds to the patient's own needs and preferences, and thus we assume that these alternatives can improve the delivery of PCC.

Methods

Ethical considerations

The Ethics Review Board of Catharina Hospital Eindhoven approved our study design. Furthermore, they decided that for this study no Medical Research Involving Human Subject approval from a Medical Ethics Committee was necessary. We informed the participants about the study and their rights as a participant in scientific research. All participants provided oral and written informed consent.

Study design

A qualitative, explorative case study approach was used to identify the touchpoints between chronically ill patients and healthcare professionals during the patient journey and to explore opportunities for digital alternatives for these touchpoints to make the patient journey more patient-centered. The consolidated criteria for reporting qualitative research (COREQ) ([22] were used as guidelines for the study design and the data analysis (S1 File).

Context

This study was conducted at the department of internal medicine of a hospital in the south of the Netherlands. The department consists of outpatient clinics for patients who suffer from illnesses such as kidney diseases, infectious diseases, vascular diseases, and metabolic diseases. The case sampling of the participants focused on typical cases of care for chronically ill patients characterized by continuous, monitoring episodes at this department. In the fall of 2021, using e-mail and telephone, the study team approached three internists at the department of internal medicine to help us select and contact potential participants. Potential participants were considered for inclusion if they visited the department of internal medicine and are treated for either diabetes, kidney failure, arteriosclerosis, or HIV.

Participants

Chronically ill patients who are treated and monitored at the department of internal medicine were asked to participate. Recruitment of these participants was carried out based on purposive sampling logic [23]. To capture a wide variety of experiences of patients who are being treated at the department of internal medicine, we focused on recruiting patients who suffer from kidney disease (kidney failure), infectious disease (HIV), vascular diseases

(arteriosclerosis), and metabolism (diabetes). Potential participants were given as much time as needed to consider whether they wished to participate and, in the case of a positive decision, were asked to reply to the internist and give consent for their contact details to be disclosed to the study team. The participants were then contacted by telephone to schedule the interview.

Data collection

The data were collected through semi-structured interviews. As a consequence of Covid-19 restrictions in the Netherlands, some of these interviews were conducted via Zoom instead of face-to face. We also collected patient characteristics such as age, gender, type of disease and how long the participants were living with their chronic illness. The interview questions were grouped according to the three phases of the patient journey: pre-service period, service period, and post-service period [13]. In each interview the same questions were asked, but not in a fixed order (S2 File). The semi-structured approach was used to obtain insights in the touchpoints in the patient journey during different periods. Additionally, we collected participants' needs, preferences and suggestions for digital alternatives for the touchpoints per period of the patient journey. The interviews were audio recorded and transcribed verbatim.

Data analysis

As our guiding principle, we used the touchpoint definition in customer journeys of Lemon & Verhoef [6]: a moment of contact between a firm or service provider and a customer at distinct points in the customer journey (p. 71). The data were analyzed using the three steps method for thematic analysis as described by Miles and Huberman [24]: 1) data reduction; 2) data display, and 3) drawing conclusions. This thematic analysis was guided by our

preliminary coding framework and based on definitions derived from the literature. The interview data were transcribed and subsequently coded. Three main categories were created to store the coded data: touchpoints, patient-centered care, and digital solutions. The definitions of these categories and their subcategories can be found in S3 File. These subcategories were later used to label the data with the relevant codes. The coding framework was continuously discussed and tested during the coding of the interviews. We started with developing a baseline patient journey, which is common practice in patient journey research [25], based on the data commonalities that were obtained from the interviews (S1 Fig). Next, we identified possible touchpoints using the touchpoint definition by Lemon & Verhoef [6]. It was then discussed whether these touchpoints could be described as healthcare encounters or device touchpoints. To obtain more detailed information about the touchpoints and about the moments at which they occurred in the patient journey, the touchpoints were grouped together according to the three periods of the patient journey. After the coding process, we systematically analyzed the touchpoints to explore whether digital alternatives that were provided by the participants could be used in the patient journey and how this could improve PCC.

Results

The study team approached twelve chronically ill patients of the department of internal medicine. Eight of them agreed to participate in this case study, none of them were from the cardiovascular patient group. Reasons for not participating that were given were: no interest in participation or not having sufficient time for the interview. The interview results will be allocated according to the three periods of a patient journey: the pre-service, service, and postservice period. The touchpoints that are experienced during these periods will be presented. Additionally, we show whether the participants are interested in digital alternatives

and which digital alternatives they would like to see implemented to improve the delivery of PCC.

Table 1. Participant characteristics.

**Participant**	**Chronic illness**	**Gender**	**Age**	**Number of years living with chronic illness**
**1**	Diabetes	Female	42	20
**2**	HIV	Male	53	16
**3**	Kidney failure	Female	78	13
**4**	Kidney failure	Female	51	1
**5**	Diabetes	Female	41	38
**6**	Kidney failure	Male	56	29
**7**	HIV	Male	41	12
**8**	HIV	Female	38	15

Table 1 provides an overview of the participant characteristics. Three of the participants were male and five of them were female. The youngest participant was 38 years old and the oldest was 78 years old (*M=50; SD=13,09*). On average, participants were living with their chronic illness for 18 years at the time of the interviews (*M=18; SD=11,26*). The interviews lasted from 20 to 55 minutes.

Pre-service period

Finding the way in and around the hospital

The interviews provided insights into touchpoints that are not directly related to the patient journey within the hospital. It was mentioned by participants that parking around the hospital can be tremendously inconvenient and time-consuming. Furthermore, finding the way around

the hospital can be difficult too, especially when one must move through the hospital in areas that have not been visited before. To reduce the unnecessary stress that participants experience about finding the way in and around the hospital, participants would like to see digital developments implemented.

"I believe that one of the areas where the hospital can be improved is the logistics. I think it would be great to be able to check in with your car license plate or pay the parking fee online" – Participant 6

"Recently, I had to walk a route in the hospital that I didn't know, so I struggled to find the fastest route. It would have been useful to have some navigation through an app for example." – Participant 7

Intake consult with the healthcare professional

In general, participants stated that they appreciate physical consults, especially when they do not yet have a strong relationship with their healthcare professional. In the intake consult the first contact between the patient and the healthcare professional takes place, a treatment plan is made together with the patient, and their treatment-related expectations are discussed. Participants indicated that they do appreciate this approach.

"I would like to see someone if I meet them for the first time. I think it truly matters whether you already have a relationship with the doctor." – Participant 8

"The first impression was positive. Everything was neatly told, for example that I will be checked every three months." – Participant 1

The participants mentioned that they usually receive several information documents, such as information leaflets, before the intake consult takes place. Some participants pointed out that digital alternatives could help them better in order to prepare the intake consult.

"Now you get an entire library of documents to read, which I did not manage to read. I think some informative clips could work better." – Participant 4

"I am a huge fan of short videos. I like to watch them way more than I like to read a folder, which I usually lose soon after I got it." – Participant 5

"I can imagine that digital solutions could work very well here, as it is a source of information that patients can approach themselves at any time." – Participant 6

A good preparation before the intake consult takes place is important to ask the healthcare professional better questions related to the treatment for their chronic illness.

"It is important to be well-informed before you meet the doctor. Otherwise, it might be unclear where the difficulties are and what things are important to take into consideration." - Participant 4

In contrast, another participant stated that she did not prepare the intake consult at all. She specified that she trusts the healthcare professional's judgment and does not want to interfere. This participant mentioned that she accepted the treatment proposals without extensive consideration.

"I let the doctor decide everything, as he knows best. I would not know how things work." – Participant 3

Check-in at the desk

Most participants indicated that they did not consider the physical check-in at the desk before their appointment as a value-added activity. However, one participant did mention that this touchpoint was value-adding.

"The people at the desk know me well, so I am able to have some small talk with them." – Participant 3

All other participants saw no or little added value of the physical check-in at the desk and addressed their interest for a digital alternative for this touchpoint.

"Checking in digitally would be fine for me. Sometimes there is quite a waiting line for the check-in desk." – Participant 1

"I think checking in at the desk is not necessary, digitally would be preferable for me, for example through an app on my telephone." - Participant 6

"Not much happens when checking in at the desk and I can imagine that you could do that digitally, you're not missing much." – Participant 7

Service period

Blood tests and checkup meetings in the hospital

In this period of the patient journey, most of the touchpoints are considered healthcare encounters. Each chronically ill patient is required to undergo periodical blood tests, which are followed up by the healthcare professionals in a checkup meeting. The regularity of these tests varies from every two months to every six months. The appointments for these blood tests can already be made digitally by the patients themselves and can usually still be scheduled on the same day. However, it is possible to walk into the blood test center without an appointment between 08:00h and 19:30h on weekdays. Participants mentioned that it was reported by the physician's assistants that the waiting line at the blood test center could go up to over an hour when no digital appointment was made.

"I have to say that the people in the hospital really said that you should make an appointment, otherwise you could be waiting for over an hour." - Participant 7 "At this point, you can make a digital appointment yourself, which I think is really convenient. I can book an appointment 45 minutes from now, it is really easy to plan it yourself." – Participant 8

One participant mentioned that when her blood sample is taken, she has to wait for up to 1.5 hours before the results are in, which are then discussed with the healthcare professional during the physical checkup meeting. The participant indicated that she would prefer a digital alternative.

"Most of the time I have to wait for 1 to 1.5 hours for my blood results. If possible, I would prefer to go home and get the results digitally." – Participant 1

Another participant stated that he usually must wait for more than two weeks to receive all the blood results. When he receives these results, he usually has to come back to the hospital once again to discuss them physically with the healthcare professional.

"I have to go to the hospital to take blood and then two weeks later I have a telephone appointment or I come by the hospital for the checkup meeting". Participant 2

Due to Covid-19 restrictions in the Netherlands almost all checkup meetings were conducted via telephone only, to limit the number of people that were visiting the hospital. This was perceived as more convenient by most of the participants because they did not have to wait in the hospital for the results.

"During Covid-19 I had my blood tested at the hospital. But then they just called me and asked me how I was doing. I liked it, that they called me." Participant 3

The digital alternative for the checkup meetings via telephone was also discussed during the interviews. Several participants indicated that they would like to have a video call with their healthcare professional.

"Especially if you know most of the routine appointments, it would be nice for me if things like that could be done online." – Participant 5

One participant indicated that she usually has two separate checkup meetings in the hospital, one appointment with her doctor and one appointment with the nurse. When the possibility of combining these two meetings via a video call was suggested, she was very positive about this.

"Yes, that would be great because then I can plan and combine these meetings in time". – Participant 1

Multiple participants elaborated further on why a video call could be a good alternative for the physical checkup meeting.

"Seeing my healthcare professional makes a conversation more personal I think. And I really appreciate that." – Participant 1

"The advantage for me would be that I can see my healthcare professional, which does provide some extra value for me." – Participant 7

To better understand the patient journey of the participants, it was discussed how they are usually notified about the telephone appointment for the checkup meeting. Many stated that the details of the telephone appointment can be seen digitally in the patient portal or in a letter that has been sent to the patient. Several participants mentioned that a timeframe is provided in which the healthcare professional will call them to discuss the blood test results, but that this is not particularly convenient for them.

"Next Monday, I have an appointment by telephone again and the time period for the call is between 11:00h and 13:30h, which is quite inconvenient." - Participant 6

"There is quite a difference in when you are called. The nurse is usually on time, or only a little late. The doctor however mostly calls at the end of the day, I guess that is when they have some spare time." - Participant 8

Some of the participants said that they would like to see improvements on the (telephone) checkup meetings to tailor it more to their needs and wishes. An example that was given is recording the telephone call and video calls with the healthcare professional. In this way, a participant can later listen or look back at the conversation, even with relatives.

"Thing like that are really important in my experience, especially if you are going to suffer from dementia for example." – Participant 5

"Yes that will work for me. If people are emotional and have to hear a story that has a lot of impact, I can imagine that not everything comes across." – Participant 7

Another suggestion that was made to help healthcare professionals in the delivery of PCC through digitalization.

"What I have seen in another hospital is that the doctor had two screens. One for himself and one for the patient to look at, and I thought that was a very good bit of openness. Then you literally see what is being written and then the doctor does not have to turn their screen." – Participant 8

Furthermore, it was also discussed whether it would be more convenient for the participants if there were broader timeslots to have an appointment in the hospital, for example after 5 p.m. Some participants stated that this could be of added value for them.

"I think a lot of people would benefit from being able to come after 5 o'clock. When you are able to include the patient's preferences, you really put a patient central." – Participant 4 "If a doctor would also see the benefit of that, then that would be great." – Participant 5

Self-monitoring

Currently, self-monitoring of one's medical condition is only applicable for patients with diabetes at the department of internal medicine. These patients can measure their own blood values and upload them digitally into the patient portal. After the values have been inserted into the patient portal, patients are called by their healthcare professional who will provide advice on, for example, how much insulin to take. Some participants suffering from other illnesses, like HIV and kidney failure, pointed out that self-monitoring is appealing for them as well.

"If it would be possible, I would definitely want to participate in that. Why not?" Participant 2

"I think this could surely be possible, especially taking my own blood pressure, weight, or something like that. I could definitely share that myself online" - Participant 6

One participant even proposed a more digitalized solution where the system itself provides an advice, instead of the healthcare professional.

"At the moment the nurse has to check my blood values and give an advice, but I think this could also be done by a system to make it somewhat faster." – Participant 1

Post-service period

Reviewing medical records

In the patient portal patients can review appointments, find general medical information about the medications they are taking, and are able to make and change appointments. Not all participants make use of the possibilities of the patient's portal. It was specified that this is because either they are unaware of the possibilities or because they prefer other methods.

"I know I can log in with my DigID, and I could probably see some things in there. But I actually have not really used it so far."- Participant 2

"I have not come that far, but I know I can see my appointments in there. And that my general practitioner has a similar system." – Participant 5

"If I am unable to come to an appointment I just call the front desk, but that does not happen often." – Participant 3

Each participant said that he or she was aware that they can investigate their medical records online. However, a lack of clarity is experienced about the sharing of medical records on the patient portal and the ability of what can and cannot be seen in these digital files.

"I checked my results twice on the patient portal when I was pregnant, but sometimes I could not see them." – Participant 1

"I did check my records on the patient portal, but I find that there is not much relevant information in there." – Participant 6

"I would like to have a better overview of every picture or scan that was once taken from me, but most of the time you can never see them after the conversation with the doctor." – Participant 8

e-Consults

One participant mentioned the use of an e-Consult via the patient portal. This entails asking a question through the portal and according to the participant this question is first reviewed at the front desk and, if it cannot be answered by one of the assistants, they will send it to the doctor.

"I do not always like calling as the person I need is not always there. I can have an eConsult, which works very well. I send a question and it will get answered by either the front desk or the doctor. This way I do not have to wait." – Participant 8

Online health platforms

The participants were asked whether they used online health platforms, like websites or Facebook pages of patient associations, for information about their chronic illness. Not all the participants made use of these platforms. Others indicated that they do look for information about the newest technologies and about other patient's experiences, but not always online.

"I do not necessarily look into these platforms on the internet, but also in magazines. I feel that you get a lot of information from the doctor in terms of results and checks. But you have to look for the newest technologies that could improve your life for yourself. I feel that the doctors expect that." – Participant 5

"I am a member of the patient association and sometimes I look into their Facebook page. There I see what other people experience, which is sometimes serious. Then I worry a bit which is not always helpful." – Participant 4

Discussion

The main aim of the present study was to ascertain the chronically ill patients' needs for digital alternatives for touchpoints between them and their healthcare professionals. Specifically, we aimed to explore which digital alternatives the patients like to see implemented into their own patient journey to help healthcare professionals providing PCC.

The patient journey of chronically ill patients

The patient journey in a hospital can be described as a three-stage process in which three different service periods are defined [13]. Though, for chronically ill patients these different periods are often not entirely applicable. Patients from the three groups that were included in this study (diabetes, kidney failure, and HIV) are likely to be treated for their illness for the rest of their lives. Most research on the topic of patient journeys has been performed on patients that have the prospect of being cured and, therefore, leave the patient journey [26]. This implies a linear journey with a clear beginning and an end. However, the findings of this study suggest that the patient journey of chronically ill patients is not a linear journey, but a continuous cycle instead (Fig 1). In this cycle, the pre-service, service, and post-service periods feed into each other. For example, a patient could perform the post-service activity of looking into the results of his blood test and use this information to ask more relevant and informed questions in a checkup meeting with the healthcare professional during the service period. This way, the journey of chronically ill patients is a continuous loop of healthcare encounters and device touchpoints. However, certain activities in a specific period may not be used during every cycle or do not apply to each patient.

Fig 1. Cyclical patient journey for chronically ill patients.

Touchpoints, digital alternatives, and PCC

In general, the patient journey starts when a patient is referred to the hospital. Commonly this happens through a referral of a general practitioner or a transfer from another hospital. This start of the patient journey is usually the same for each patient, irrespective of their illness. Table 2 presents an overview of the touchpoints we identified in each period of the patient

journey, the suggested digital alternative for each touchpoint, and which dimension of PCC this digital alternative would improve, based on dimensions identified by Gerteis et al. [4].

Table 2. Touchpoints, alternatives, and improved PCC dimensions in the pre-service, service and post-service period.

**Pre-service period**	**(Digital) Alternatives**	**Improved PCC dimension**
Intake consult	Recording the meeting	• Respect for patients' values and preferences<br>• Communication between patient and professionals<br>• Involvement of family and friends<br>• Transition and continuity
Information leaflets/brochures/folders	Present information through short video clips	• Respect for patients' values and preferences<br>• Communication between patient and professionals
Check-in	Check-in screen	Respect for patients' values and preferences<br>Coordination of care
**Service period**	**(Digital) Alternatives**	**Improved PCC dimension**
Blood tests	Making digital appointments beforehand	• Respect for patients' values and preferences<br>• Coordination of care<br>• Communication between patient and professionals
Checkup meetings	Getting a video call instead of physical checkup meeting	• Respect for patients' values and preferences<br>• Coordination of care<br>• Involvement of family and friends
Selfmonitoring	Uploading results into the patient portal	• Respect for patients' values and preferences<br>• Coordination of care<br>• Communication between patient and professionals
**Post-service period**	**(Digital) Alternatives**	**Improved PCC dimension**

Administrative actions	Being able to see nonmedical information on a digital platform. As well as the possibility of booking e-Consults	- Respect for patients' values and preferences<br>- Coordination of care
Review of medical records	Being able to see medical results and context of those results	- Respect for patients' values and preferences
Contacting the hospital for questions	Being able to have eConsults with healthcare professionals	- Respect for patients' values and preferences<br>- Coordination of care<br>- Communication between patient and professionals
Online Health Platforms	Being able to see information and experiences from similar patients	- Respect for patients' values and preferences<br>- Emotional support and alleviation of fear and anxiety

The check-in process in medical settings is occasionally already done through a digital interface at a screen in the waiting area to improve patient experience and to potentially reduce patient waiting time [27]. In this way, the check-in process can be perceived as more efficient and made significantly faster when implementing this digital alternative. When adjusting the physical check-in into a digital check-in before an appointment it is no longer a healthcare encounter, but it can be considered a device touchpoint. This device touchpoint is in accordance with the majority of the patients' expressed needs and relates to a different coordination of care. Furthermore, most patients addressed their interest in a digital alternative for the information documents they receive before the intake consult takes place. By providing this information digitally, in the form of short video clips, healthcare professionals meet the majority of the patients' needs and wishes and thus provide PCC. Table 2 provides an overview of possible alternatives for touchpoints and the affected PCC dimensions in the pre-service period.

Regarding the service period, the most important findings were that most of the patients prefer digital check-up meetings and the option of digitally self-monitoring one's condition. During the Covid-19 pandemic, the

physical check-up meetings mostly have been replaced by telephone calls. Patients perceived this as more convenient because they did not have to wait in the hospital for the results of their blood sample. Our findings suggest that a digital alternative for telephone calls, for example video calls, could also be implemented in the patient journey in order to make it more patient-centered. This is in line with the findings of a previous study [28] that found that most patients are satisfied with video calls and prefer them as an alternative to physical meetings. Patients felt more comfortable with video visits than office visits and expressed a preference for receiving future serious news via video visit, because they could be in their own supportive environment [28]. So, instead of calling the patient, the check-up meeting could be done through a video call. Patients value the option of being able to see their healthcare professional and believe that it is also valuable for the healthcare professional to see the patient. Furthermore, patients are positive about the option of recording the video call. This would make it possible for a patient to listen or look back at the conversation, even with relatives, at a convenient moment. According to the findings of this study, another digital alternative for a touchpoint in the service period that could be implemented is to make it possible for patients to monitor their own medical status. This involves that the patient uploads medical values in a digital system, which are checked by the healthcare professional who can then provide medical advice based on these values. Currently, self-monitoring is being implemented in the treatment of diabetes patients. Selfmonitoring of one's medical status is a form of telemonitoring. A previous study [29] found that patients comply with telemonitoring programs and the use of technologies. Therefore, home telemonitoring of chronic diseases seems to be a promising patient management approach that produces accurate and reliable data, empowers patients, influences their attitudes and behaviors, and potentially improves their medical conditions. With technology moving at a rapid pace, non-diabetic patient groups may also benefit from self-monitoring practices in the future as well. Digitally self-monitoring was found to be very appealing for the patients, which shows their willingness to take control over their own medical

process. In Table 2 these possible alternatives for touchpoints and the affected PCC dimensions in the service period are presented.

Concerning the post-service period, the most important finding was that most of the chronically ill patients experienced a lack of clarity about the sharing of medical records on the patient portal and the ability of what can(not) be seen in these digital files. Therefore, patients do not often use the patient portal. This finding is similar to a previous study [30] that presents knowledge barriers of how and when to use patient portals is one of the many perceived barriers by patients to use these portals. Considering that there is a need for more knowledge and awareness about possibilities patient portals offer, it could be of added value when the healthcare professional demonstrates the patient possibilities and benefits of the patient portal. Moreover, healthcare professionals could also raise more awareness about eConsults. e-Consults can be an addition to the communication channels that the hospital already makes use of, such as e-mail and telephone, which would allow the hospital to be more varied in their contact options. Additionally, it could be made possible that the healthcare professional has the option to video call the patient following the question that was asked in the e-Consult. A video call is generally perceived as a more personal way of communicating compared to a regular phone call [28]. An additional advantage of the use of e-consults is that it can reduce the waiting time for the patients that contact the hospital by phone. Patients are increasingly looking for information about their disease on their own. They are not only searching on sites supported by healthcare professionals, but also across the internet and on social media like Facebook and Twitter. One of the areas where they are looking for information is on platforms where patients with the same chronic illness share their experiences. Sometimes these platforms are unregulated, but some are controlled by patient associations. The use of online health platforms could be managed by the hospital to make sure that the patient obtains reliable medical information. As can be seen in Table 2 these possible alternatives for touchpoints in the post-service period can improve a variety of PCC dimensions.

Limitations, recommendations, and future research

Clearly, there are limitations of the present study. The primary limitation of this study is the generalizability of the results, as with a small sample size caution must be applied. The aim was to include multiple patients from four distinct patient groups: HIV, diabetes, kidney failure, and cardiovascular diseases. However, it proved to be very hard to find patients suffering from cardiovascular diseases who were willing to participate. Additionally, mainly middle-aged patients, between 35-60 years, were included and the sample size lacked patients younger than 35 years old. This could make the results of this study biased towards the middle-age group. Younger and older patients could potentially provide other interesting insights and suggestions of digitalization and the patient journey. Besides, according to our findings chronically ill patients have an essentially different patient journey compared to nonchronically ill patients. Our findings suggest a continuous cycle, rather than a linear patient journey, for chronically ill patients. Therefore, the results may not be entirely generalizable towards other (non-chronically ill) patient groups.

The recommendations that can be derived from this study apply to different levels: the patient level, the healthcare professional level, and the hospital management level. On the patient level, it is advisable to keep communicating with the patients that are experiencing the touchpoints firsthand. Their feedback is extremely valuable and often generalizable to a larger part of the patient population, as chronically ill patients often have similar wishes and needs.

Additionally, there is a noteworthy number of patients that have stable conditions that would like to assess their medical status. For example, take their weight, measure blood pressure, or even blood values if possible. Improved technology could make this possible for more patients in the future, as now only diabetes patients actively perform self-monitoring activities. However, for most patients, it is not clear what digital possibilities are already available for them through the patient portal. Therefore, there is a challenge for hospitals to communicate and educate about the existing digital possibilities.

On the level of healthcare professionals, it is also important that they are aware of the existing digital possibilities. Furthermore, as is the case for patients, healthcare professionals need to be trained to use the digital platform in a

consistent way. It is not preferable to have some healthcare professionals using the platform very extensively, while others are rarely making use of it.

On the hospital management level, it would be advisable to install digital check-in screens, as most of the patients do not see the added value of checking in at the desk but do so of asking questions. Consequently, employees at the desk will have more time to answer questions that patients may have. For the hospital management, this transition to digital check-in screens would also be beneficial, when keeping the increasing shortage of medical personnel in mind.

In terms of PCC, it would be desirable to perform a more extensive study of the current experiences that patients have with digitalization. Common patterns and experiences could be identified, and possible future digital applications could be based on the current experiences of the patients and their actual needs and preferences. Ideally, this would be done across the entire hospital to be able to obtain different insights from various perspectives. The concept of visualization each interactive touchpoint that the patient experiences as they navigate the care continuum, e.g., patient journey mapping, is a relatively novel practice [26] and therefore there is no fixed or widely accepted method of establishing the patient journey.

This might also be a cause for the lack of adoption of patient journey mapping in the healthcare industry [31]. Therefore, it might be interesting to capture the patient journey in a different manner, for example through questionnaires or observations or a method that solely focuses on the digital side of the patient journey.

Conclusion

Literature showed that a patient journey can be divided into three distinct periods: pre-service period, service period, and post-service period. However, the empirical data showed that these periods are cyclical instead of a linear process for chronically ill patients. In this cyclical patient journey, digitalization can help put the wishes and needs of the patients at the center of care. Digital alternatives to all kinds of touchpoints within the patient journey can make the journey less of a

burden and increase the level of communication between the patient and the healthcare professional. Ultimately, digitalization can help support the delivery of PCC, as it helps patients to flow more efficiently through their patient journey while establishing higher levels of communication with their healthcare professionals.

References

1. Reynolds A. Patient-centered care. Radiologic Technology. 2009;81(2): 133-147. Available from: http://www.radiologictechnology.org/content/81/2/133.long
2. Epstein RM, Street RL. The values and value of patient-centered care. Annals of Family Medicine. 2011;9(2): 100-103. doi: 10.1370/afm.1239.
3. Peters VJT, Meijboom BR, Bunt JEH, Bok LA, van Steenbergen MW, de Winter JP, et al. Providing person-centered care for patients with complex healthcare needs: A qualitative study. PLoS ONE. 2020;15(11): e0242418. doi: 10.1371/journal.pone.0242418.
4. Gerteis M, Edgman-Levitan S, Walker JD, Stoke DM, Cleary PD, Delbanco TL. What patients really want. Health Management Quarterly. 1993;15(3): 2-6. Available from: https://pubmed.ncbi.nlm.nih.gov/10129190/
5. Gualandi R, Masella C, Viglione D, Tartaglini D. Exploring the hospital patient journey: What does the patient experience? PLoS ONE. 2019;14(1): 1-15. doi: 10.1371/journal.pone.0224899.
6. Lemon KN, Verhoef PC. Understanding customer experience throughout the customer journey. Journal of Marketing. 2016;80(6): 69-96. doi: 10.1509/jm.15.0420.

7. Carayon P, Wooldridge A, Hoonakker P, Schoofs A, Kelly MM. SEIPS 3.0: Humancentered design of the patient journey for patient safety. Applied Ergonomics. 2020;84: 103033. doi: 10.1016/j.apergo.2019.103033.

8. Homburg C, Jozić D, Kuehnl C. Customer experience management: Toward implementing an evolving marketing concept. Journal of the Academy of Marketing Science. 2017;45(3): 377-401. doi: 10.1007/s11747-015-0460-7.

9. McCarthy S, O'Raghallaigh P, Woodworth S, Lim YL, Kenny LC, Adam F. An integrated patient journey mapping tool for embedding quality in healthcare service reform. Journal of Decision Systems. 2016;25: 354-368. doi: 10.1080/12460125.2016.1187394.

10. Walford S, Allison SP, Gale EAM, Tattersall RB. Self-monitoring of blood glucose improvement of diabetic control. The Lancet. 1978;17: 732-735. doi: 10.1016/s0140-6736(78)90855-3.

11. Meyer MA. COVID-19 pandemic accelerates the need to improve online patient engagement practices to enhance patient experience. Journal of Patient Experience. 2020;7(5): 657-664. doi: 10.1177/2374373520959486.

12. Andino JJ, Guduguntla V, Weizer A, Roberts WW, Wittmann D, Miller D, et al. Examining the value of video visits to patients in an outpatient urology clinic. Urology. 2017;110: 31-35. doi: 10.1016/j.urology.2017.07.050.

13. Rosenbaum MS, Otalora ML, Ramírez GC. How to create a realistic customer journey map. Business Horizons. 2017;60(1): 143-150. doi: 10.1016/j.bushor.2016.09.010.

14. Mosher ZA, Hudson PW, Lee SR, Perez JL, Arguello AM, McGwin G, et al. Check-in kiosks in the outpatient clinical setting: Fad or the future? Southern Medical Journal.

2020;113(3): 134-139. doi: 10.14423/SMJ.0000000000001078.

15. Ramaswamy A, Yu M, Drangsholt S, Ng E, Culligan PJ, Schlegel PN, Hu JC. Patient satisfaction with telemedicine during the COVID-19 pandemic: Retrospective cohort study. Journal of Medical Internet Research. 2020;22(9): 1-9. doi: 10.2196/20786.
16. Gardner MR, Jenkins SM, O'Neil DA, Wood DL, Spurrier BR, Pruthi S. Perceptions of video-based appointments from the patient's home: A patient survey. Telemedicine and E-Health. 2015;21(4): 281-285. doi: 10.1089/tmj.2014.0037.
17. Ross SE, Lin CT. The effects of promoting patient access to medical records: A review. Journal of the American Medical Informatics Association. 2003;10(2): 129138. doi: 10.1197/jamia.m1147.
18. Otte-Trojel T, de Bont A, Rundall TG, & van de Klundert J. How outcomes are achieved through patient portals: A realist review. Journal of the American Medical Informatics Association. 2014;21(4): 751-757. doi: 10.1136/amiajnl-2013-002501.
19. Meyer MA. Mapping the patient journey across the continuum: Lessons learned from one patient's experience. Journal of Patient Experience. 2019;6(2): 103-107. doi: 10.1177/2374373518783763.
20. van der Eijk M, Faber MJ, Aarts JW, Kremer JA, Munneke M, Bloem BR. Using online health communities to deliver patient-centered care to people with chronic conditions. Journal of Medical Internet Research. 2013;15(6): e115. doi: 10.2196/jmir.2476.
21. Granström E, Wannheden C, Brommels M, Hvitfeldt H, Nyström ME. Digital tools as promoters for person-centered care practices in chronic care? Healthcare professionals' experiences from rheumatology care. BMC Health Services Research.

2020;20: 1108. doi: 10.1186/s12913-020-05945-5.

22. Tong A, Sainsbury P, Craig J. Consolidated criteria for reporting qualitative research (COREQ): A 32-item checklist for interviews and focus groups. International Journal for Quality in Health Care. 2007;19(6): 349-357. doi: 10.1093/intqhc/mzm042.
23. Patton MQ. Qualitative research and evaluation methods. Thousand Oaks: Sage Publications; 2002.
24. Miles MB, Huberman AM, Saldaña J. Qualitative data analysis: A methods sourcebook. Thousand Oaks: Sage Publications; 2014.
25. Alkandari M, Ryan K, Hollywood A. The experiences of people living with peripheral neuropathy in Kuwait — A process map of the patient journey. Pharmacy (Basel, Switzerland). 2019;7(3): 127. doi: 10.3390/pharmacy7030127.
26. Joseph AL, Kushniruk AW, Borycki EM. Patient journey mapping: Current practices, challenges and future opportunities in healthcare. Knowledge Management & E-Learning: An International Journal. 2020;12(4): 387-404. doi: 10.34105/j.kmel.2020.12.021
27. Mahmood A, Wyant DK, Kedia S, Ahn S, Powell MP, Jiang Y, Bhuyan SS. Selfcheck-in kiosks utilization and their association with wait times in emergency departments in the United States. The Journal of Emergency Medicine. 2020;58(5), 829-840. doi: 10.1016/j.jemermed.2019.11.019.
28. Powell RE, Henstenburg JM, Cooper G, Hollander JE, Rising KL. Patient perceptions of telehealth primary care video visits. The Annals of Family Medicine. 2017;(3): 225229. doi: 10.1370/afm.2095.
29. Paré G, Jaana M, Sicotte C. Systematic review of home telemonitoring for chronic diseases: The evidence base. Journal of the American Medical Informatics Association. 2007;14(3): 269-277. doi: 10.1197/jamia.M2270.

30. Griffin A, Skinner A, Thornhill J, Weinberger M. Patient Portals: Who uses them? What features do they use? And do they reduce hospital readmissions? Applied Clinical Informatics. 2016;7: 489-501. doi: 10.4338/ACI-2016-01-RA-0003.

31. Sijm-Eeken M, Zheng J, Peute L. Towards a lean process for patient journey mapping – A case study in a large academic setting. Studies in Health Technology and Informatics. 2020;270: 1071-1075. doi: 10.3233/SHTI200326.

The never-ending patient journey of chronically ill patients: A qualitative case study

ORIGINALITY REPORT

49%	11%	7%	45%
SIMILARITY INDEX	INTERNET SOURCES	PUBLICATIONS	STUDENT PAPERS

PRIMARY SOURCES

1	Submitted to Tilburg University<br>Student Paper	40%
2	research.tilburguniversity.edu<br>Internet Source	2%
3	d-scholarship.pitt.edu<br>Internet Source	1%
4	sigma.nursingrepository.org<br>Internet Source	1%
5	www.ncbi.nlm.nih.gov<br>Internet Source	1%
6	www.science.gov<br>Internet Source	1%
7	Submitted to A.T. Still University - Missouri<br>Student Paper	<1%
8	www.jmir.org<br>Internet Source	<1%
9	journals.plos.org<br>Internet Source	<1%

10	Submitted to NHS Business Leadership Academy<br>Student Paper	<1 %
11	research-api.cbs.dk<br>Internet Source	<1 %
12	link.springer.com<br>Internet Source	<1 %
13	Submitted to University of Greenwich<br>Student Paper	<1 %
14	www.theseus.fi<br>Internet Source	<1 %
15	Janet R. McColl-Kennedy, Hannah Snyder, Mattias Elg, Lars Witell, Anu Helkkula, Suellen J. Hogan, Laurel Anderson. "The changing role of the health care customer: review, synthesis and research agenda", Journal of Service Management, 2017<br>Publication	<1 %
16	www.kmel-journal.org<br>Internet Source	<1 %
17	www.proton-pump.com<br>Internet Source	<1 %
18	platcovid.com<br>Internet Source	<1 %
19	revistes.ub.edu<br>Internet Source	

<1%

20	diabetes.jmir.org<br>Internet Source	<1%
21	ww2.health.wa.gov.au<br>Internet Source	<1%
22	V. Bartali, L. Brandl, J. Karreman, L. van Velsen. "Making good decisions is hard when you are at your worst: Investigating the degree of users' decision-making in an e-Mental health service", Research Square Platform LLC, 2022<br>Publication	<1%
23	jamanetwork.com<br>Internet Source	<1%
24	bmcgeriatr.biomedcentral.com<br>Internet Source	<1%
25	coek.info<br>Internet Source	<1%
26	ore.exeter.ac.uk<br>Internet Source	<1%

Exclude quotes	Off	Exclude matches	Off
Exclude bibliography	On		

The never-ending patient journey of chronically ill patients: A qualitative case study

GRADEMARK REPORT

FINAL GRADE

/0

GENERAL COMMENTS

Instructor

PAGE 1

PAGE 2

PAGE 3

PAGE 4

PAGE 5

PAGE 6

PAGE 7

PAGE 8

PAGE 9

PAGE 10

PAGE 11

PAGE 12

PAGE 13

PAGE 14

PAGE 15

PAGE 16

PAGE 17

PAGE 18

PAGE 19

PAGE 20

PAGE 21

PAGE 22

PAGE 23

PAGE 24

PAGE 25

PAGE 26

PAGE 27

PAGE 28

PAGE 29

PAGE 30

PAGE 31

PAGE 32
