# Supplementary material for: The never-ending patient journey of chronically ill patients: A qualitative case study on touchpoints in relation to patient-centered care
Source: PLoS One. 2023 May 17;18(5):e0285872. doi: 10.1371/journal.pone.0285872 (PMC10191281; doi:10.1371/journal.pone.0285872)
Supplement: Attachment — Submitted filename: Response to Reviewers.docx [file pone.0285872.s004.docx]

**Journal requirements**

**Q1 Please ensure that your manuscript meets PLOS ONE's style requirements, including those for file naming. The PLOS ONE style templates can be found at**

**https://journals.plos.org/plosone/s/file?id=wjVg/PLOSOne_formatting_sample_main_body.pdf and**

**https://journals.plos.org/plosone/s/file?id=ba62/PLOSOne_formatting_sample_title_authors_affiliations.pdf**

R: We have carefully checked the PLOS ONE’s style requirements, including those for file naming.

**Q2 In your Data Availability statement, you have not specified where the minimal data set underlying the results described in your manuscript can be found. PLOS defines a study's minimal data set as the underlying data used to reach the conclusions drawn in the manuscript and any additional data required to replicate the reported study findings in their entirety. All PLOS journals require that the minimal data set be made fully available. For more information about our data policy, please see http://journals.plos.org/plosone/s/data-availability.**

**Upon re-submitting your revised manuscript, please upload your study’s minimal underlying data set as either Supporting Information files or to a stable, public repository and include the relevant URLs, DOIs, or accession numbers within your revised cover letter. For a list of acceptable repositories, please see http://journals.plos.org/plosone/s/data-availability#loc-recommended-repositories. Any potentially identifying patient information must be fully anonymized.**

**Important: If there are ethical or legal restrictions to sharing your data publicly, please explain these restrictions in detail. Please see our guidelines for more information on what we consider unacceptable restrictions to publicly sharing data: http://journals.plos.org/plosone/s/data-availability#loc-unacceptable-data-access-restrictions. Note that it is not acceptable for the authors to be the sole named individuals responsible for ensuring data access.**

**We will update your Data Availability statement to reflect the information you provide in your cover letter.**

R: We have now specified in the cover letter where the minimal data set underlying the results described in our manuscript can be found. Thanks for updating our Data Availability statement accordingly.

**Editor**

**Q3. Just as observed by the reviewers, the methods can be improved. I suggest the following**

R: Thank you for your comments. We will go over your suggestions point-by-point below.

**Methods**
**Q4. Even though the authors mentioned in the abstract that data was collected from 8 participants, this important information is not included in the methods in main text.**

R: We have now included the number of participants in our manuscript.

*C: The data were collected through eight semi-structured interviews.* (Page 11).

**Q5. Normally qualitative research requires between 9 to 24 in-depth interviews. As also highlighted by other reviewers, motivate the selection of just eight participants for the interviews; that is too little and likely to threaten the validity and generalizability of studies’ results. See the following reference for ideal sample size.**

**Vasileiou, K., Barnett, J., Thorpe, S. et al. Characterising and justifying sample size sufficiency in interview-based studies: systematic analysis of qualitative health research over a 15-year period. BMC Med Res Methodol 18, 148 (2018).** [**https://doi.org/10.1186/s12874-018-0594-7**](https://doi.org/10.1186/s12874-018-0594-7)

**Monique Hennink, Bonnie N. Kaiser, Sample sizes for saturation in qualitative research: A systematic review of empirical tests, Social Science & Medicine, Volume 292, 2022,** [**https://doi.org/10.1016/j.socscimed.2021.114523**](https://doi.org/10.1016/j.socscimed.2021.114523)**.**

R: We agree that the number of respondents of our study is relatively low. However, given the fact that we conducted an exploratory study, previous studies have shown that a limited amount of interviews can be sufficient in order to get a reliable sense of thematic exhaustion and variability. We encountered data saturation after 7 interviews, as no new themes or touchpoints emerged from the data gathered between interview seven and interview eight. We incorporated one of the suggested references and an additional reference to elaborate on our reasoning and justification for interviewing eight participants. Please see our manuscript accordingly.

*C: The sample size aim was to include three patients of each of the patient groups. This number was based on research that proves that data saturation can be achieved with 9 to 17 interviews [25]. Generally, reaching saturation, meaning new interviews do not yield new data on the interview topics, is considered sufficient for validity [26]. In the case of exploratory studies, a limited amount of interviews can be sufficient [26] in order to get a reliable sense of thematic exhaustion and variability within our data set.* (Page 10).

Additional references:

25. Hennink M, Kaiser BN. Sample sizes for saturation in qualitative research: A systematic review of empirical tests. Social Science & Medicine. 2021;292: 114523. <https://doi.org/10.1016/j.socscimed.2021.114523>.

26. Guest G, Bunce A, Johnson L. How many interviews are enough? An experiment with data saturation and variability. Field Methods. 2006;18(1): 59-82.

<https://doi.org/10.1177/1525822X05279903>.

**Q6. As observed by the reviewer, semi-structured interview is not always the best method for exploratory study design. Unstructured interviews such as in-depth interviews would have been the most appropriate for of data collection method for your study.**

R: We agree with the reviewer that a semi-structured interview is not always the best method for exploratory study design. We have added a reference to strengthen our reasoning for the use of semi-structured interviews and, for next studies, will carefully consider the use of unstructured interviews for exploratory study design.

*C:* *A semi-structured interview lends itself well to qualitative research, as it is an efficient way to gather rich data and it allows for the creation of new insights into the studied case [27].* (Page 11).

Additional reference:

27. Verleye K. Designing, writing-up and reviewing case study research: An equifinality perspective. Journal of Service Management. 2019;30(5): 549-576.

<https://doi.org/10.1108/JOSM-08-2019-0257>.

**Q7. Your ethics statement should come after the data analysis section, just before the results section. You should also include the ethic reference number**

R: We have amended the order of the Methods section; the ethical considerations section now follows the validity and reliability section. Furthermore, we have added the ethic reference number. Please see our manuscript accordingly.

*C. The Ethics Review Board of Catharina Hospital Eindhoven approved our study design (nWMO-2021.055).* (Page 15).

**Reviewer #1**

**Q8. Thank you for this opportunity to review your manuscript in which you describe the patient journey of chronically ill patients as cyclical and never-ending and could be augmented by digitalisation of touchpoints. Overall, this is interesting research, and your study could help inform policy and clinical practice. Your paper could be improved in the following ways.**

R: We thank the reviewer for the compliments and will go over her comments point-by-point below.

**Background.
Q9. The literature is well-used to support your research question. A lot of what you talk about in terms of patient-centric care and digitalisation can be related to the Chronic Care Model (1) in which the touchpoints you refer to can be considered ‘productive interactions’. Did you have a reason for not using this model in your research? If so, I suspect you will need to justify why you didn’t use the model. In my opinion its use gives strength to a lot of what you did.**

R: We aimed to explore which digital alternatives patients would like to see implemented into their patient journey to help healthcare professionals providing patient-centered care. We have considered using the Chronic Care Model in the design of the study, but belief our focus is more in alignment with the seven dimensions affecting PCC as introduced by Gerteis et al. (1993). We now acknowledge the existence of the Chronic Care Model in the manuscript.

*C:* *Although we acknowledge the eHealth Enhanced Chronic Care Model, as proposed by Gee et al. [22], which places chronic care in the context of the community where the person will receive care, we are convinced that the work by Gerteis et al. [4] provides a more comprehensive view on how digitalization can improve dimensions of PCC.* (Page 8).

Additional reference:

22. Gee PM, Greenwood DA, Paterniti DA, Ward D, Miller LMS. The eHealth enhanced chronic care model: A theory derivation approach. Journal of medical Internet research. 2015;17(4): 4-5.

https://[doi:10.2196/jmir.4067](https://doi.org/10.2196/jmir.4067).

**Methods.**
**Q10. You say you used purposive sampling but what you describe sounds more like convenience sampling, e.g., you had access to certain departments in a hospital and those departments covered certain health issues which in turn influenced who responded to your invitation to participate in interviews. In other words, you did interviews with people who were available (convenience) rather than your first choice of interviews (purposive). Please consider modifying your description of the type of sampling you did.**

R: We agree that convenience sampling technique is a better description of our sampling strategy. We modified the manuscript accordingly.

*C: Recruitment of these participants was carried out based on convenience sampling logic.* (Page 10).

**Q11. I would prefer to see you create a table of the interview questions and insert it in the body of the manuscript than for you to have it as supplementary information. That way a time-poor reader doesn’t have to follow links to interview questions. Also, please consider adding the demographic items in the table so that it’s clear when and how you got that information, i.e., that you didn’t get demographic data from a third party (the clinic).**

R: We agree with the reviewer that it is better to include the interview questions into the manuscript itself as opposed to providing it as a supplementary file. Please see our manuscript accordingly (Page 11 and 12).

**Q12. There are certain qualities in qualitative data, especially in interviews, that need to be unpacked in the methods section, e.g., data saturation, why you only completed eight interviews and what satisfied you that you could stop at eight, what constituted your case study (you say it was a case study but nowhere do you say why it was a case study and how interviews within a case study are useful to answer your research question). Take a look at this article by Morse (2) about ways to strengthen qualitative research so that you can indicate what you did to mitigate the weaknesses/limitations of a set of interviews. You may also find this article useful to explain your decision to interview the eight people you interviewed and the role of the interviewees’ context (3). You need to describe your methods decisions in such a way that another researcher can replicate your methods. At the moment, I’m not confident that your study can be replicated.(4)**

R: We now include more detail on data saturation, why we only completed eight interviews, and what constituted our case study. Please see our manuscript accordingly.

*C: The sample size aim was to include three patients of each of the patient groups. This number was based on research that proves that data saturation can be achieved with 9 to 17 interviews [25]. Generally, reaching saturation, meaning new interviews do not yield new data on the interview topics, is considered sufficient for validity [26]. In the case of exploratory studies, a limited amount of interviews can be sufficient [26] in order to get a reliable sense of thematic exhaustion and variability within our data set.* (Page 10).

Additional references:

25. Hennink M, Kaiser BN. Sample sizes for saturation in qualitative research: A systematic review of empirical tests. Social Science & Medicine. 2021;292: 114523. <https://doi.org/10.1016/j.socscimed.2021.114523>.

26. Guest G, Bunce A, Johnson L. How many interviews are enough? An experiment with data saturation and variability. Field Methods. 2006;18(1): 59-82.

<https://doi.org/10.1177/1525822X05279903>.

**Q13. I think that the coding table should go into the methods section with a brief statement about how you derived the codes.**

R: We have added the coding table into the data analysis section of the Methods section with a brief statement on how we derived the codes. Please see the manuscript accordingly. (Page 15, 16 and 17).

*C: The coding framework was continuously discussed and tested during the coding of the interviews, which is in alignment with the main principle that Miles et al. endorse and entails that codes should ‘have some conceptual and structural unity. Codes should relate to one another in coherent, study-important ways; they should be part of a unified structure’.* (Page 13).

**Findings.
Q14. You indicate that the demographic profile of the interviewees is skewed to older people in this section and in the limitations. Is it possible for you to get a report on the demographics of the clinic patients who form the context of your research? All you need to get is a demographic profile of ages from the three clinics from which you sourced your interviews to be able to say if self-selection for interviews was because people with those conditions are usually older or older people tend to self-select for research participation. There is some literature to support the latter conclusion, and it shouldn’t be hard to find, assuming that is what you discovered in your findings.**

R: We thank the reviewer for the suggestion to add a demographic profile of ages of the patient groups to support our statement that the age of the interviewees is skewed to middle-aged patients. We have added this information in the form of Table 1 (Demographic profile of ages) on page 10 and 11. We incorporated two additional references to substantiate our statement. Please see the manuscript accordingly.

*C: Table 1 provides an overview of the demographic profile of these four patient groups.* (page 10).

*Table 1. Demographic profile of patient groups at department of Internal Medicine.* (page 10).

| *Patient group* | *Total number of patients (N)* | *Age (M, SD)* | *Age (Min-Max)* |
| --- | --- | --- | --- |
| *Arteriosclerosis* | *12* | *62.0 (11.65)* | *42-76* |
| *Diabetes* | *1535* | *58.4 (22.29)* | *17-95* |
| *HIV* | *615* | *47.4 (18.45)* | *19-88* |
| *Kidney failure* | *598* | *71.6 (20.84)* | *19-97* |

*However, Saphner et al. [37] found that patients younger than age 65 are more likely to participate in clinical studies compared to patients 65 or older. Hutchins et al. endorse this finding, as they conclude that the most consistent and largest disparity in study participation pertains to age [38]. Our findings are based on mostly middle-aged participant, but younger and older patients could potentially provide other interesting insights and suggestions of digitalization and the patient journey.* (Page 31).

Additional references:

37. Saphner T, Marek A, Homa JK, Robinson L, Glandt N. Clinical trial participation assessed by age, sex, race, ethnicity, and socioeconomic status. Contemp Clin Trials. 2021 Apr;103: 106315.

https://doi: 10.1016/j.cct.2021.106315.

38. Hutchins LF, Unger JM, Crowley JJ, Coltman CA Jr, Albain KS. Underrepresentation of patients 65 years of age or older in cancer-treatment trials. N Engl J Med. 1999 Dec 30;341(27): 2061-7.

https://doi: 10.1056/NEJM199912303412706.

**Q15. There are too many quotes. This leaves the reader feeling that they need to do some analysis – the analysis is your job. The use of multiple quotes for one point creates questions in the reader’s mind – did you finish coding the quotes, were these the only quotes for this point, does this mean that if you used one quote for a point that only one person gave you a quote for that point?**

R: We agree with the reviewer that we have made abundant use of quotes to illustrate our findings. We have carefully read our Results section and have reduced the number of quotes. Initially there were 42 quotes in the Results section, in the revised manuscript we kept 31 quotes. Please see our manuscript accordingly.

**Q16. Table 2 about touchpoints belongs in the findings section. It’s a very nice table and can be used to complement the thematic narrative.**

R: We thank the reviewer for the compliments on the table about the touchpoints, which we use to substantiate and clarify the link between our empirics and the literature. We therefore believe that it is placed appropriately in the Discussion section.

**Q17. I like the figure that demonstrates the cyclic nature of the touchpoints, but it’s not clear in the findings that you saw this. Placement of this figure into the findings section (not the discussion section) should be reconsidered.**

R: We believe that Figure 1 comes into its own in the Discussion section, because this figure was created on the basis of our interpretation of the study results rather than a readily available internal process. In the Results sections, we do not want to elaborate on interpreting the results, we want to leave that for the Discussion section.

**Q18. Because you can't make the raw data freely available you should consider not using supplementary files and rather insert the content of your supplementary files into the manuscript where they below.**

R: Thank you for sharing these thoughts with us. We have decided to upload the minimal data set to replicate the study to a stable, public repository. In addition, we have included the content of the supplementary files into the manuscript where they belong when appropriate. Please see the manuscript accordingly.

**Discussion
Q19. There are some very good descriptions of the findings in the discussion section. Some of these descriptions belong on the findings section. A good discussion should (1) summarise the key findings (done, but repetitive), (2) compare and contrast the findings with what’s already in the literature (partially done), (3) indicate how the research question/s was/were answered (could be done more clearly). It is in this section that you can draw strongly on the Chronic Care Model (and the associated literature about digitisation of ‘productive interactions’) to strengthen the points you want to make from your findings.**

R: Thank you for your suggestions. We have now rewritten (and removed) some parts of the discussion. We now also build on the Chronic Care Model as per your suggestion. Please see the manuscript accordingly.

C: *This finding is in accordance with the eHealth Enhanced Chronic Care Model of Gee et al. who propose that access to and control over personal health data due to policy changes is needed to provide the consumer more autonomy. Some health care organizations have had positive experiences with open access for consumers, including the provider notes [22].* (Page 28-29).

*Digitally providing information is also in alignment with the findings of Gee et al. [22], who argue that the role of the community to provide support for patient engagement or activation and for self-management should be expanded to include online community and health-related social networks.* (Page 29-30).

Additional reference:

22. Gee PM, Greenwood DA, Paterniti DA, Ward D, Miller LMS. The eHealth enhanced chronic care model: A theory derivation approach. Journal of medical Internet research. 2015;17(4): 4-5.

https://[doi:10.2196/jmir.4067](https://doi.org/10.2196/jmir.4067).

**Limitations.
Q20. You can’t use quantitative research measures to critique the value and truth of qualitative research. It is more appropriate to talk about transferability, trustworthiness, data saturation, triangulation and member describing the strengths and weaknesses of your research methods. Use Morse’s article to help you do that. Use Malterud’s article to help justify the number of interviews you did. Indicate what types of contexts can and should use your research findings, i.e., similar to the context of your research.**

R: We did not intend to only use quantitative research measures to critique our qualitative work. We have been more careful in our wording and build our argument on the article of Morse.

*C: Transferability entails the extent to which it can be applied in other contexts and studies [31]. Morse et al. [31] elaborates that in qualitative research the application of the findings to another situation or population is achieved through de-contextualization and abstraction of emerging concepts and theory which should be the prerogative of the original investigator. However, in our study the findings can hardly be interpreted separate from its context. Moreover, caution must be applied with results based on a small sample size.* (Page 30-31).

Additional reference:

31. Morse JM. Critical analysis of strategies for determining rigor in qualitative inquiry. Qualitative Health Research. 2015;25(9): 1212-1222.

<https://doi.org/10.1177/1049732315588501>

**References.
Q21. The list of references appears to be focused well on the research being reported, and articles from a range of years are used. I have referred below to articles that you should use to strengthen your manuscript.**

R: We thank the reviewer for her suggested references and have incorporated those to strengthen our manuscript. Please see the manuscript accordingly.

**General proofreading issues.
Q22. There is a tendency to use the odd word oddly, e.g., In the abstract you say, ‘These digital alternatives consisted of video … viewing their own medical status in a digital manner’ Do you mean ‘digital format’? The former is ambiguous.**

R: We agree with the reviewer and changed the wording accordingly. We also changed this elsewhere in the manuscript.

*C: These digital alternatives consisted of video calls, digitally checking in before a physical appointment, digitally self-monitoring one’s medical condition and personally uploading monitoring results into the patient portal, and viewing their own medical status in a digital format.* (Page 3).

*Therefore, it might be interesting to capture the patient journey using a different approach****.*** (Page 32).

**Q23. When the subject of a sentence is singular/plural you make the rest of the sentence plural/singular, e.g., ‘Each patient is unique and requires treatments that suits …’ Suits should be suit because at the subject of the sentence is ‘each patient’ implies plural. Confusing I know. I recommend that you get someone to go through the manuscript and clean these minor errors up for you.**

R: We agree with the reviewer that we have been sloppy in terms of singular/plural wording. We cleaned these minor errors up with the help of an English native speaker.

*C: Each patient is unique and requires treatments that suit their situation in a patient-centered way.* (Page 5).

**Q24. People can’t be ‘it’, e.g., ‘Some of these healthcare encounters that are currently taking place physically at the hospital could also be performed by the patient itself.’ Delete ‘itself’.**

R: We fully agree that people cannot be referred to as it. We have carefully read the manuscript and have deleted those kind of references. Please see the manuscript accordingly.

**Q25. My references for this review:
1. Gee PM, Greenwood DA, Paterniti DA, Ward D, Miller LMS. The eHealth enhanced chronic care model: a theory derivation approach. Journal of medical Internet research. 2015;17(4).
2. Morse JM. Critical analysis of strategies for determining rigor in qualitative inquiry. Qualitative health research. 2015;25(9):1212-22.
3. Malterud K, Siersma VD, Guassora AD. Sample size in qualitative interview studies: guided by information power. Qualitative health research. 2016;26(13):1753-60.
4. Coiera E, Ammenwerth E, Georgiou A, Magrabi FJJotAMIA. Does health informatics have a replication crisis? 2018;25(8):963-8.**

R: We thank the reviewer for the suggested references and have incorporated those, when appropriate, in our manuscript.

**Reviewer #2**

**Q26. These are some comments to improve the current study**

R: We thank the reviewer for the comments to improve our manuscript and will go over the comments point-by-point below.

**Q27. Major revision of the title – based on the intent – multiple points of contact and PCC with physical /face to face and technology.**

R: We agree with the reviewer that the title of our manuscript could be more clearly tailored to the intent of our study, and therefore we updated it. Please see the manuscript accordingly.

*C: The never-ending patient journey of chronically ill patients: A qualitative case study on touchpoints in relation to patient-centered care* (Page 1 and 2).

**Q28. Basically this study is about whether integrating digital technology in the continuity and continuum of care would lead to a quality outcomes. The study is more like pilot /feasibility study.**

R: This study indeed can be viewed more like a pilot/feasibility study about whether integrating digital technology in the continuity and continuum of care can lead to quality outcomes for chronically ill patients. We have changed some wording in the manuscript to highlight this.

**Q29. Figure 1- Revisit and align with the intent of the study, that is the continuum of care -disagree that it is a cyclical process it is rather a non-linear process -depending on their condition and the aspect of self-monitoring need to be somehow factored in.**

R: There is certainly something to be said that the patient journey of chronically ill patients can be described as a non-linear process. However, when describing something as non-linear it means that it does not progress or develop from one stage tot the next stage in a logical way. This is not the case in our study, because the patient actually does progress from one stage to another. Instead, we argue that the patient journey of chronically ill patients is a cyclical process in which a series of events happens again and again in a somewhat similar and logical sequence. In the Discussion section we briefly address this. Please see the manuscript accordingly.

*C: For chronically ill patients it is common that a series of touchpoints happens again and again in a somewhat similar and logical sequence.* (Page 25).

**Q30. Sup File 1 - The "stop" in the figure contradicts the cyclical process/ continuum -see also the result section which goes beyond the health system.**

R: The ‘stop’ in figure S1 “Visualization of the baseline patient journey” refers to the baseline patient journey of a patient in a hospital. This journey entails a clear beginning and end (as the patient is cured), and, therefore, there is a ‘stop’ in this figure. The cyclical continuum in Figure 1 refers to the patient journey of chronically ill patients. Since they are likely to be treated for the rest of their lives, their patient journey does not entail a clear ending. Additionally, in the patient journey of chronically ill patients events tend to happen again and again in a somewhat similar sequence. Without this clear ending and with these recurring events, we argue that the patient journey of this specific patient group is cyclical instead of linear.

**Q31. In the research design/method section -Appears to be more towards a survey method using structured interview approach and does not qualify for a qualitative research with phenomenological method of data analysis.**

R: The applied study method is a qualitative, exploratory case study approach. The consolidated criteria for reporting qualitative research (COREQ) were used as guidelines for the study design and the data analysis. We actually do believe that it therefore qualifies for a qualitative research with phenomenological method of data analysis.

**Q32. A descriptive exploratory research design using a survey method through structured interviews is evident with the listed questionnaire items in the supplementary file.**

R: The listed interview items in the supplementary file are now added to the main manuscript (Page 11 and 12). These questions were asked during the interviews, however not in a fixed order which is common practice in semi-structured interviews. We used these questions as a guideline for our interviews, but we also asked follow-up questions in relation to the answers of the participants, per the nature of semi-structured interviews. Consequently, we made use of a semi-structured interview design.

**Q33. Provide some detail on the reliability and validity / trustworthiness, etc. irrespective of research design and methods used.**

R: We thank the reviewer for this suggestion and agree that we should provide some details on these topics. That is why we now have added a section about validity and reliability into the Methods section in which we elaborate on these topics. This new section is placed between the data analysis section and the ethical considerations section. Additionally we elaborate a bit on the topic of transferability in the Discussion section.

*C: To establish validity and reliability of our data, several measures have been taken [30]. To improve the internal validity, the interviewed patients were initially contacted by their healthcare professionals to ask whether they would take part in this research. The study and its purpose were explained. To further increase reliability, the semi-structured interviews have been conducted in Dutch, the native language of the participants, so that no language barriers exist. Additionally the member checking method was applied. This method entails to check that the transcription was correctly done by returning the transcript of the interview to the participants. [31]. The transcripts were returned to the eight participants. We received no comments and corrections*. (Page 15).

*C: Transferability entails the extent to which it can be applied in other contexts and studies [31]. Morse et al. [31] elaborates that in qualitative research the application of the findings to another situation or population is achieved through de-contextualization and abstraction of emerging concepts and theory which should be the prerogative of the original investigator. However, in our study the findings can hardly be interpreted separate from its context. Moreover, caution must be applied with results based on a small sample size.* (Page 30 and 31).

Additional references:

30. Golafshani, N. Understanding reliability and validity in qualitative research. *The Qualitative Report*. 2003:*8*(4): 597-606.

<https://doi.org/10.46743/2160-3715/2003.1870>.

31. Morse JM. Critical analysis of strategies for determining rigor in qualitative inquiry. Qualitative Health Research. 2015;25(9): 1212-1222.

<https://doi.org/10.1177/1049732315588501>.

**Q34. Lastly, the concept of consumerism with touch points in their journey leans towards a concrete rather than abstract construct which weakly align with qualitative approach.**

R: We understand the concern of the reviewer that in the context of consumerism touchpoints can be considered concrete, perhaps even physical constructs rather than abstract constructs. However, speaking of a “patient journey’ as a metaphor is abstract in itself and given the fact that it was not clear which touchpoints were present in the patient journey, we first had to conduct an exploratory, qualitative approach to identify the touchpoints present in this ‘journey’. Future research can build on our study and use a quantitative approach to further uncover the potential of touch points.

*C: Therefore, it might be interesting to capture the patient journey using a different approach, for example through questionnaires or observations or a method that solely focuses on the digital side of the patient journey. In addition, future research could build on our identified touchpoints and measure their effectiveness in relation to PCC provision.* (Page 33 and 33).

**Reviewer #3**

**Q35. The manuscript “the never-ending patient journey of chronically ill patients: A qualitative case study” is of great importance and interest. The manuscript has some serious originality concerns as the similarity index is 49% which is exceptionally high. The authors have made no such disclosures if it is part of some previous project or extracted from the dissertation. Without such information, it is difficult for me to comment on the other aspects of the manuscript. Therefore, I recommend the rejection of the manuscript at this stage.**

R: Thank you for acknowledging the importance of our manuscript. We have carefully looked at the similarity index and acknowledge that 40% of our content stems from the Master’s thesis. Part of the study has been published as a Master thesis. We have the approval to republish the data from the University where the Master thesis has been written. We also cite the source in our article. We belief this should not be an issue, since this is original work by one of the authors of this study. We hope that by clarifying the comment raised, and highlighting this in the cover letter, you will be able to comment on the other aspects of our manuscript.

Additional reference:

40. Dibbets, FH. The digital side of healthcare: How digitalizing the patient journey can increase patient-centered care. [master’s thesis]. Tilburg (NL): Tilburg University; 2022.
