# Supplementary material for: The never-ending patient journey of chronically ill patients: A qualitative case study on touchpoints in relation to patient-centered care
Source: PLoS One. 2023 May 17;18(5):e0285872. doi: 10.1371/journal.pone.0285872 (PMC10191281; doi:10.1371/journal.pone.0285872)
Supplement: Attachment — Submitted filename: Response to Reviewers.docx [file pone.0285872.s005.docx]

**Editor**

**Q1. A major concern for two of the three reviewers were issues related to the methodology used in this study, and I concur with their comments. This section should be clearly written, with a clear description of each of the sub-sections for ease of replication. The appropriate approach for this study is a semi-structured in-depth interview and this should pull through in your data collection and analysis sections. Actually what the authors described in the data collection section is a face-to-face (or virtual) individual in-depth interviews which were audio recorded.**

R: Thank you for your comments. We agree that the Methods section should be clearly written for ease of replication. We have conducted a major overhaul of the Methods section for improvements in terms of transparency and replication purposes. Please see the relevant changes for this comment below.

*C. A qualitative, exploratory case study design was used to identify the touchpoints between chronically ill patients and healthcare professionals during the patient journey and to explore opportunities for digital alternatives for these touchpoints to make the patient journey more patient-centered. Because the topic of this study is in its formative stage, we conducted qualitative research in the form of a case study. Case study research designs have different purposes, namely exploratory, explanatory, and descriptive, or a combination of those purposes [23]. The design of the current study can be defined as exploratory, as it explores the patient journey of chronically ill patients, their attitudes towards that journey, and the possible digital alternatives provided by these patients for the encountered touchpoints during their journey. We used a case study approach to generate an in-depth understanding of a complex issue in its real-life context, which lends itself well to capture information on ‘how’, ‘what’, and, ‘why’ questions [24]. An advantage of conducting research in this manner is that it provides the opportunity to study a topic in its real-life context; this can contribute to understanding whether digital alternatives for touchpoints make the patient journey of chronically ill patients more patient-centered. The consolidated criteria for reporting qualitative research (COREQ) [25] were used as guidelines for the study design and the data analysis (S1 File).* (Page 9 and 10).

*The study data were gathered using a cross-sectional approach, meaning that we collected data from different individuals at a single point in time. The data were collected through eight semi-structured interviews. The semi-structured nature of the interviews allowed us to make sure that important topics were covered, while leaving room for the participants to provide their story regarding their patient journey [30]. A semi-structured interview is relevant for exploratory research, as it is an effective way to gather rich data and it allows for the creation of new insights into the case under study [23]. As a consequence of Covid-19 restrictions in the Netherlands, some of these interviews were conducted via Zoom instead of face-to face. We also collected patient characteristics such as age, gender, type of disease and how long the participants were living with their chronic illness. The interview questions were grouped in advance according to the three phases of the patient journey: pre-service period, service period, and post-service period [13]. A case study approach is particularly useful when there is a need to obtain an in-depth appreciation of an issue, in which qualitative interviews are preferred over quantitative methods alone [24]. Therefore, we are convinced that our data collection approach is aligned with the aim of our study. In each interview the same questions were asked, but not in a fixed order (Table 2). The semi-structured approach was used to obtain insights in the touchpoints in the patient journey during different periods. In addition, we collected participants’ needs, preferences, and suggestions for digital alternatives for the touchpoints per period of the patient journey. The interviews were audio recorded and transcribed verbatim.*

*We also collected relevant documentation that was open to the public (e.g. information leaflets, invitation letters), and internal documentation of the department of internal medicine (e.g. planning schemes, medical protocols). These collected documents provided valuable information in terms of touchpoints encountered by chronically ill patients during their patient journey.* (Page 13).

*The data were analyzed using the three steps method for thematic analysis as described by Miles et al. [31]: 1) data reduction, 2) data display, and 3) drawing conclusions. This is a systematic data reduction process building on, among others, the reading of transcripts, document summaries, codification of text segments, generation of themes and categories, and identification of relationships [31]. We employed this deductive, thematic analysis approach since we used a coding framework for analysis that was based on concepts and definitions derived from the literature. While exploratory case studies probe into and shed light on what is essentially unknown, they should be guided by a specific purpose that frames the research [23]. The deductive codes were useful in both the segmentation and coding phase of the data analysis. Three main categories were created to store the coded data: touchpoints, patient-centered care, and digital solutions. The definitions of these categories and their subcategories can be found in Table 3. These (sub)categories were later used to label the data with the relevant codes. The coding framework was continuously discussed and tested during the coding of the interviews, which is in alignment with the main principle that Miles et al. [31] endorse and entails that codes should ‘have some conceptual and structural unity. Codes should relate to one another in coherent, study-important ways; they should be part of a unified structure’ [31].*

*We started with developing a baseline patient journey, which is common practice in patient journey research [32], based on the data commonalities that were obtained from the interviews (S1 Fig). Next, as our guiding principle, we used the touchpoint definition in customer journeys of Lemon & Verhoef [6] to identify touchpoints in the patient journey: a moment of contact between a firm or service provider and a customer at distinct points in the customer journey (p. 71). It was then discussed within the research team whether these touchpoints could be described as healthcare encounters or device touchpoints. To obtain more detailed information about the touchpoints and about the moments at which they occurred in the patient journey, the touchpoints were grouped together according to the three periods of the patient journey, as identified by Rosenbaum et al. [13]. After the coding process, we systematically analyzed the touchpoints to explore whether digital alternatives that were provided by the participants could be used in the patient journey and how this could improve PCC, using the dimensions identified by Gerteis et al. [4].* (Page 16-17).

Additional references:

23. Verleye K. Designing, writing-up and reviewing case study research: An equifinality perspective. Journal of Service Management. 2019;30(5): 549-576. doi: 10.1108/JOSM-08-2019-0257.

24. Crowe S, Cresswell K, Robertson A, Huby G, Avery A, Sheikh A. The case study approach. BMC Medical Research Methodology. 2011;11(100): 1-9. doi: 10.1186/147-2288-11-100.

**Q2. Other issue raised was the adequacy of the eight purposive samples used in this study, for which the authors responded with the argument of reaching saturation and cited Guest et al., 2006. How many interviews are enough? An experiment with data saturation and variability.**

**However, the excerpt below from this paper says otherwise "Although the idea of saturation is helpful at the conceptual level, it provides little practical guidance for estimating sample sizes, prior to data collection, necessary for conducting quality research."  "Purposive samples still need to be carefully selected, and twelve interviews will likely not be enough if a selected group is relatively heterogeneous, the data quality is poor, and the domain of inquiry is diffuse and/or vague. Likewise, you will need larger samples if your goal is to assess variation between distinct groups or correlation among variables. For most research enterprises, however, in which the aim is to understand common perceptions and experiences among a group of relatively homogeneous individuals, twelve interviews should suffice." Also refer to:**

**Monique Hennink, Bonnie N. Kaiser, Sample sizes for saturation in qualitative research: A systematic review of empirical tests, Social Science & Medicine, Volume 292, 2022,** [**https://doi.org/10.1016/j.socscimed.2021.114523**](https://doi.org/10.1016/j.socscimed.2021.114523)

**Vasileiou, K., Barnett, J., Thorpe, S. et al. Characterising and justifying sample size sufficiency in interview-based studies: systematic analysis of qualitative health research over a 15-year period. BMC Med Res Methodol 18, 148 (2018).** [**https://doi.org/10.1186/s12874-018-0594-7**](https://doi.org/10.1186/s12874-018-0594-7)

R: We have conducted a major overhaul of the subsection ‘Participants’ of the Methods section to describe the adequacy of the eight purposive samples used in this study in more detail. We have included the suggested references in our manuscript. Please see the manuscript accordingly.

*C. Chronically ill patients who are treated and monitored at the department of internal medicine were asked to participate. Recruitment of these participants was carried out based on convenience sampling logic [26]. To capture a wide variety of experiences of patients who are being treated at the department of internal medicine, we focused on recruiting patients who suffer from kidney disease (kidney failure), infectious disease (HIV), vascular diseases (arteriosclerosis), and metabolic diseases (diabetes). Table 1 provides an overview of the demographic profile of ages of these four patient groups. Potential participants were given as much time as needed to consider whether they wished to participate and, in the case of a positive decision, were asked to reply to the internist and give consent for their contact details to be disclosed to the study team. The participants who gave consent were then contacted by telephone to schedule the interview. The sample size aim was to include at least three patients of each patient group, so that we would conduct twelve interviews in total. This number of interviews was based on previous research that proves that data saturation, meaning new interviews do not yield new data on the interview topics, can be achieved with 9 to 17 interviews [27, 28]. Eight patients agreed to participate in this study, despite our continued efforts to include more patients. Although the number of participants is relatively small, a limited amount of interviews can be sufficient in the case of exploratory studies in order to get a reliable sense of thematic exhaustion and variability of the data [27, 28]. In addition, research has indicated that sample size should not be considered alone but be embedded in the more encompassing examination of data adequacy [29]. This implies that sample size numbers in qualitative research are not unimportant, but should be extended to terms of adequate amounts of evidence, adequate variety in kinds of evidence, and adequate interpretive status of evidence. Given our comprehensive data collection, consisting of semi-structured interviews and document analysis, we are convinced that, despite our relatively small number of interviews, we were able to present adequate variety and interpretation of our evidence. By combining the information from the interviews and document analysis with the theoretical framework, we were able to draw a comprehensive image of the patient journey for the patient groups included in our study. For instance, when an interviewee mentioned a specific touchpoint, we verified this touchpoint with our collected documents and interview transcripts to ensure that this touchpoints was in accordance with the baseline patient journey we developed. This approach strengthened us in our belief that we were able to present convincing evidence. This conviction also arose during the data analysis when we observed that data saturation happened after seven interviews, as no new themes emerged from the data gathered between interview seven and interview eight. This is in accordance with previous research that demonstrated that saturation can be achieved in a narrow range of interviews [27].* (Page 10, 11 and 12).

*In this study we used a qualitative, exploratory study design to develop an initial understanding of the patient journey of chronically ill patients and possible digital improvements of the touchpoints in this patient journey. We acknowledge that our sample size is smaller than recommended by the findings of multiple studies [27, 28]. The aim was to include multiple patients from four distinct patient groups: HIV, diabetes, kidney failure, and cardiovascular diseases. However, it proved to be very hard to find patients suffering from cardiovascular diseases who were willing to participate.* (Page 36 and 37).

Additional references:

27. Hennink M, Kaiser BN. Sample sizes for saturation in qualitative research: A systematic review of empirical tests. Social Science & Medicine. 2021;292: 114523. doi: 10.1016/j.socscimed.2021.114523.

29. Vasileiou K, Barnett J, Thorpe S, Young T. Characterising and justifying sample size sufficiency in interview-based studies: Systematic analysis of qualitative health research over a 15-year period. BMC Medical Research Methodology. 2018;18(148): doi: 10.1186/s12874-018-0594-7.

**Q3. Reviewer #1 made very useful comments that were not adequately addressed. See the following: “Table 2 about touchpoints belongs in the findings section. It’s a very nice table and can be used to complement the thematic narrative."  I suggest including Table 2 in your result section and discuss it in further in your discussion section.**

R: We apologize for not adequately addressing the useful comments of reviewer #1 about the placement of Table 2. In our initial manuscript, this was Table 2. In our first revised manuscript it was labeled as Table 5, though it was still placed in the Discussion section. We now have placed Table 5 at the end of the Results section and discuss it in further detail in our discussion section. Please see our manuscript accordingly.

C. *Table 5 presents an overview of the touchpoints we identified in each period of the patient journey, the suggested digital alternative for each touchpoint, and which dimension(s) of PCC this digital alternative would improve, based on the dimensions identified by Gerteis et al. [4].* (Page 29).

*We will now discuss in more detail the findings of the current study that were listed in Table 5 with the findings from the literature.* (Page 32).

**Q4. "There are certain qualities in qualitative data, especially in interviews, that need to be unpacked in the methods section, e.g.,... what constituted your case study (you say it was a case study but nowhere do you say why it was a case study and how interviews within a case study are useful to answer your research question). ...You need to describe your methods decisions in such a way that another researcher can replicate your methods. At the moment, I’m not confident that your study can be replicated.(4)"**

**Refer to the following paper on how to conduct a case study:**

**Crowe, S., Cresswell, K., Robertson, A. *et al.* The case study approach. *BMC Med Res Methodol* 11, 100 (2011).** [**https://doi.org/10.1186/1471-2288-11-100**](https://doi.org/10.1186/1471-2288-11-100)

R. We conducted a major overhaul of our Methods section and provide more detail on what constituted our case study, why it was a case study, and how interviews were useful to answer our research question. These adjustments will add to the ease of replication of our study. We have used the suggested reference and included it in the reference list.

C. *A qualitative, exploratory case study design was used to identify the touchpoints between chronically ill patients and healthcare professionals during the patient journey and to explore opportunities for digital alternatives for these touchpoints to make the patient journey more patient-centered. Because the topic of this study is in its formative stage, we conducted qualitative research in the form of a case study. Case study research designs have different purposes, namely exploratory, explanatory, and descriptive, or a combination of those purposes [23]. The design of the current study can be defined as exploratory, as it explores the patient journey of chronically ill patients, their attitudes towards that journey, and the possible digital alternatives provided by these patients for the encountered touchpoints during their journey. We used a case study approach to generate an in-depth understanding of a complex issue in its real-life context, which lends itself well to capture information on ‘how’, ‘what’, and, ‘why’ questions [24]. An advantage of conducting research in this manner is that it provides the opportunity to study a topic in its real-life context; this can contribute to understanding whether digital alternatives for touchpoints make the patient journey of chronically ill patients more patient-centered.* (Page 9 and 10).

*We took the healthcare provision for chronically ill patients characterized by continuous, monitoring episodes, provided at the department of internal medicine of a hospital in the south of the Netherlands, as our case. The department consists of outpatient clinics for patients who suffer from (chronic) illnesses such as kidney diseases, infectious diseases, vascular diseases, and metabolic diseases. The case sampling of the participants focused on typical cases of care for chronically ill patients characterized by continuous, monitoring episodes at this department. In the fall of 2021, using e-mail and telephone, the study team approached three internists at the department of internal medicine to help us select and contact potential participants. Potential participants were considered for inclusion if they visited the department of internal medicine and are treated for either kidney failure, HIV, arteriosclerosis, or diabetes.* (Page 10).

*The study data were gathered using a cross-sectional approach, meaning that we collected data from different individuals at a single point in time. The data were collected through eight semi-structured interviews. The semi-structured nature of the interviews allowed us to make sure that important topics were covered, while leaving room for the participants to provide their story regarding their patient journey [30]. A semi-structured interview is relevant for exploratory research, as it is an effective way to gather rich data and it allows for the creation of new insights into the case under study [23]. As a consequence of Covid-19 restrictions in the Netherlands, some of these interviews were conducted via Zoom instead of face-to face. We also collected patient characteristics such as age, gender, type of disease and how long the participants were living with their chronic illness. The interview questions were grouped in advance according to the three phases of the patient journey: pre-service period, service period, and post-service period [13]. A case study approach is particularly useful when there is a need to obtain an in-depth appreciation of an issue, in which qualitative interviews are preferred over quantitative methods alone [24]. Therefore, we are convinced that our data collection approach is aligned with the aim of our study. In each interview the same questions were asked, but not in a fixed order (Table 2). The semi-structured approach was used to obtain insights in the touchpoints in the patient journey during different periods.* (Page 12 and 13).

Additional reference:

24. Crowe S, Cresswell K, Robertson A, Huby G, Avery A, Sheikh A. The case study approach. BMC Medical Research Methodology. 2011;11(100): 1-9. doi: 10.1186/147-2288-11-100.

30. Saunders M, Lewis P, Thornhill A. Research methods for business students. Harlow: Pearson Education; 2009.

**Q5. "...did you finish coding the quotes, were these the only quotes for this point, does this mean that if you used one quote for a point that only one person gave you a quote for that point?"**

R. When we used a single quote in our Results section, this does not indicate that only one participant gave a quote for that point. We then used this quote because, in our opinion, this quote illustrated and clarified the findings of the interviews. In response to the previous comments made by Reviewer #1 in the first round of revision, we removed 11 quotes. Initially, there were 42 quotes in the Results section, in the previous revised manuscript we kept 31 quotes to illustrate our findings.

**Q6. Even though the authors claimed to have used thematic analysis, it is unclear what approach was used for the analysis. Deductive or inductive approach, or both?**

R. We have elaborated on the approach we used for the thematic analysis in the subsection ‘Data analysis’ of the Methods section. Please see our manuscript accordingly.

*C. The data were analyzed using the three steps method for thematic analysis as described by Miles et al. [31]: 1) data reduction, 2) data display, and 3) drawing conclusions. This is a systematic data reduction process building on, among others, the reading of transcripts, document summaries, codification of text segments, generation of themes and categories, and identification of relationships [31]. We employed this deductive, thematic analysis approach since we used a coding framework for analysis that was based on concepts and definitions derived from the literature. While exploratory case studies probe into and shed light on what is essentially unknown, they should be guided by a specific purpose that frames the research [23]. The deductive codes were useful in both the segmentation and coding phase of the data analysis.* (Page 16).

Additional reference:

23. Verleye K. Designing, writing-up and reviewing case study research: An equifinality perspective. Journal of Service Management. 2019;30(5): 549-576. doi: 10.1108/JOSM-08-2019-0257.

**Q7.** **The authors were given a chance to rectify these flaws, however, these have not been adequately addressed in the revised submission. For these reasons, I cannot recommend this manuscript for publication in the current form.**

R. We apologize for not adequately rectifying the flaws in our revised submission. We hope that the improvements we have currently made make the manuscript now suitable for publication.

**Q8. Please review your reference list to ensure that it is complete and correct. If you have cited papers that have been retracted, please include the rationale for doing so in the manuscript text, or remove these references and replace them with relevant current references. Any changes to the reference list should be mentioned in the rebuttal letter that accompanies your revised manuscript. If you need to cite a retracted article, indicate the article’s retracted status in the References list and also include a citation and full reference for the retraction notice.**

R. We have now carefully reviewed our reference list to ensure that it is complete and correct. We have included additional references based on the suggestions by the editor and the reviewer(s). Please see the manuscript accordingly.

**Reviewer #2**

**Q9. Though the topic is sound and relevant. Currently the study has methodological flaws and require a careful thought through approach whether it qualify for a qualitative approach.**

R. We thank reviewer #2 for acknowledging that the topic of our manuscript is sound and relevant. We hope that by addressing the comments of the editor we also have improved the methodological flaws and comments concerning the qualitative approach mentioned by Reviewer #2.

**Reviewer #3**

**Q10.  The Authors performed adequate revisions and the scientific contribution of the work has improved. As a result of this, I recommend accepting the manuscript for publication in PLOS One.**

R: We thank reviewer #3 for acknowledging that we made adequate revisions and for recommending to accept our manuscript for publication in PLoS ONE.
