# Supplementary material for: The never-ending patient journey of chronically ill patients: A qualitative case study on touchpoints in relation to patient-centered care
Source: PLoS One. 2023 May 17;18(5):e0285872. doi: 10.1371/journal.pone.0285872 (PMC10191281; doi:10.1371/journal.pone.0285872)
Supplement: Attachment — Submitted filename: Dear Plos One.docx [file pone.0285872.s006.docx]

Dear Plos One,

According tot he abstract I thought I could review the article of Peters V et al.

But after reading the manuscript twice I have to admit that I have to resign on what is asked. I have nothing to add or comment on the manuscript.

Sorry for the delay

Yours L Bok, pediatrician
