# Supplementary material for: The never-ending patient journey of chronically ill patients: A qualitative case study on touchpoints in relation to patient-centered care
Source: PLoS One. 2023 May 17;18(5):e0285872. doi: 10.1371/journal.pone.0285872 (PMC10191281; doi:10.1371/journal.pone.0285872)
Supplement: Attachment — Submitted filename: Response to Reviewers.docx [file pone.0285872.s008.docx]

Reviewer 3

**Q1. I have reviewed your manuscript and have compiled a list of major revisions based on the suggestions provided.**

R. We thank the reviewer for his extensive comments. We have addressed your comments in more detail below.

**Q2. Page 3: Line 41-44: Authors may consider rephrasing this sentence for more clarity: "Participants were included if they had visited the department of internal medicine and had received treatment for either diabetes, kidney failure, arteriosclerosis, or HIV."**

R. We thank the reviewer for this suggestion and have now rephrased the sentence for more clarity. In addition, we now also present the chronic illnesses in alphabetical order throughout the manuscript.

*C.* *Participants were included if they had visited the department of internal medicine and had received treatment for either arteriosclerosis, diabetes, HIV, or kidney failure.* (Page 3).

**Q3. Page 10: Line 218: I would like to suggest a small change to the section on the sampling method.
In the manuscript, the authors have referred to the recruitment method as "convenience sampling logic". I believe that this may cause some confusion among readers. It would be more accurate to simply state that convenience sampling was used to select participants and that purposive sampling was used to target specific chronic illnesses in order to capture a wide variety of experiences.
I think this clarification would make the sampling method more understandable to readers and help address any potential confusion that may arise.**

R. We thank the reviewer for this suggestion and agree that this clarification can prevent any potential confusion that may arise. We have changed the manuscript accordingly.

*C. Chronically ill patients who received treatment and are monitored at the department of internal medicine were asked to participate. First, convenience sampling logic was used to identify potential participants [26]. Second, purposive sampling was used to target specific chronic illnesses to capture a wide variety of experiences of patients who are being treated at the department of internal medicine, with a focus on patients who suffered from infectious disease (HIV), kidney disease (kidney failure), metabolic diseases (diabetes), and vascular diseases (arteriosclerosis).* (Page 10 and 11).

**Q4. Page 11: Line 222: In the participant recruitment section, the authors mentioned that potential participants were given as much time as needed to consider whether they wished to participate. However, it would be helpful to readers if you could clarify the specific time frame given to participants to decide.
I would suggest adding a sentence to this section that specifies the amount of time given to participants, for example, "Participants were given one week to consider whether they wished to participate in the study." This would provide readers with a clear understanding of the time frame used in the recruitment process.**

R. We have now added more detail to specify the amount of time given to participants to cinsider whether they wished to participate. In the Netherlands, two weeks is considered an appropriate time frame to consider potential participation in scientific research. Please see the manuscript accordingly.

*C.* *Potential participants were given two weeks to consider whether they wished to participate and, in the case of a positive decision, were asked to reply to the internist and give consent for their contact details to be disclosed to the study team.* (Page 11).

**Q5. Page 12: Line 252: In the current presentation of the table, the headings and the order of the columns are as follows: "Patient Group, number of patients, Average age, Standard deviation, minimum age, maximum age". I would like to suggest reordering the columns and changing the heading "Patient Group" to "Patient Group by Disease" for improved clarity. Additionally, it would be helpful if you could shuffle the data according to the following sequence:
"Number of patients, Patient Group by Disease, Average age, Standard deviation, minimum age, maximum age"
Finally, I would suggest placing the table in the section of the manuscript where it is being referred to, for easier reference by the readers.**

R. We have changed the order of the data of Table 1 according to the suggested sequence. Please see the manuscript accordingly.

*C.*

| ***Number of patients*** | ***Patient group by disease*** | ***Average age*** | ***Standard deviation*** | ***Minimum age*** | ***Maximum age*** |
| --- | --- | --- | --- | --- | --- |
| *12* | *Arteriosclerosis* | *62.01* | *11.65* | *42* | *76* |
| *1535* | *Diabetes* | *58.43* | *22.29* | *17* | *95* |
| *615* | *HIV* | *47.75* | *18.45* | *19* | *88* |
| *598* | *Kidney failure* | *71.62* | *20.84* | *19* | *97* |

(Page 12).

**Q6. In the current presentation, the figures have been reported with varying numbers of decimal places. For example, some figures have been reported with one decimal place, while others have been reported with two decimal places. This inconsistency may cause confusion for readers. To improve the clarity and consistency of your manuscript, I suggest using a consistent style of reporting figures. Specifically, I recommend reporting all figures with two decimal places to ensure consistency and clarity throughout the manuscript.**

R. We agree with the reviewer that we have been inconsistent in terms of decimal places. We have now ensured that we consistently apply two decimal places throughout the manuscript.

C. *The study team approached twelve chronically ill patients of the department of internal medicine. Eight of them agreed to participate in the present study, none of them were from the cardiovascular patient group. Reasons for not participating that were given were no interest in participation or not having sufficient time for the interview. Table 2 provides an overview of the participant characteristics. Three of the participants were male and five of them were female. The youngest participant was 38 years old and the oldest was 78 years old (M=50.00 years; SD=13.09). On average, participants were living with their chronic illness for 18 years at the time of the interviews (M=18.00 years; SD=11.26). The interviews lasted from 20 to 55 minutes.* (Page 12).

**Q7. Page 12: Line 255: Given the qualitative and exploratory nature of the study, the focus was primarily on understanding the experiences, perceptions, and attitudes of individuals rather than on collecting quantitative data at a single point in time. Therefore, instead of mentioning the cross-sectional approach, the authors may want to emphasize the qualitative and exploratory aspects of the study, and how these informed the data collection and analysis methods used. This can provide readers with a clearer understanding of the purpose and scope of the study.**

R. We agree that emphasizing the qualitative and exploratory aspects provides the readers with a clearer understanding of the purpose and scope of our study.

*C. We conducted eight semi-structured interviews which allowed us to make sure that important topics were covered, while leaving room for the participants to provide their story regarding their patient journey [30]. Given the qualitative and exploratory nature of our case study, the focus was primarily on understanding the experiences, perceptions, and attitudes of chronically ill patients during their patient journey. The semi-structured interview approach is relevant for exploratory research, as it is an effective way to gather rich data and it allows for the creation of new insights into the case under study [23].* (Page 13).

**Q8. Page 12: Line 262: Given that Zoom is a widely-used video conferencing platform, it may be helpful for readers to have a more specific reference to the company and location in the manuscript. Therefore, we suggest that the authors include the company's name, Zoom Video Communications, Inc., and its headquarters location, San Jose, California, USA, when referring to the video conferencing software used in the study. This can enhance the clarity and transparency of the methods section for readers.**

R. We thank the reviewer for this suggestion and now have added a more specific reference in the manuscript.

*C. As a consequence of Covid-19 restrictions in the Netherlands in 2021, five interviews were conducted via Zoom [31] instead of face-to face.* (Page 13).

Additional reference:

31. Zoom Video Communications, Inc. Zoom. Version 5.8.3 [software]. San Jose: Zoom Video Communications, Inc; 2021. Available from: <https://zoom.us/>

**Q9. Page 13: Line 266: I noticed that the authors mentioned the benefits of a case study approach in both the previous section and this section. It may be beneficial to merge similar information in one place to avoid repeating information.**

R. To avoid repeating the information related to the benefits of a case study approach we grouped these statements together in the Study design paragraph. Please see our manuscript accordingly.

*C. A qualitative, exploratory case study design was used to identify the touchpoints between chronically ill patients and healthcare professionals during the patient journey and to explore opportunities for digital alternatives for these touchpoints to make the patient journey more patient-centered. Because the topic of this study is in its formative stage, we conducted qualitative research in the form of a case study. Case study research designs have different purposes, namely exploratory, explanatory, and descriptive, or a combination of those purposes [23]. The design of the current study can be defined as exploratory, as it explores the patient journey of chronically ill patients, their attitudes towards that journey, and the possible digital alternatives provided by these patients for the encountered touchpoints during their journey. We used a case study approach to generate an in-depth understanding of a complex issue in its real-life context, which lends itself well to capture information on ‘how’, ‘what’, and ‘why’ questions [24]. An advantage of conducting research in this manner is that it provides the opportunity to study a topic in its real-life context; this can contribute to understanding whether digital alternatives for touchpoints make the patient journey of chronically ill patients more patient-centered. The consolidated criteria for reporting qualitative research (COREQ) [25] were used as guidelines for the study design and the data analysis (S1 File).* (Page 9 and 10).

**Q10. Page 13: Line 268: While it is important to align the data collection approach with the aim of the study, the reader can draw their own conclusion from the reported data. Therefore, omitting the statement in the conclusion section may be more appropriate.**

R. We have now omitted the statement to ensure that readers can draw their own conclusion from the reported data. Please see the manuscript accordingly.

**Q11. Page 13: Line 270: I have noticed that the phrase "the semi-structured approach" appears multiple times in the methods section of your manuscript. While it is important to describe the methodology thoroughly, I believe that some of the repetitions may be unnecessary and could disrupt the text's flow. Therefore, I suggest you review the methods section and consider consolidating some of the descriptions or finding alternative ways to convey the same information without repeating the exact same phrase multiple times.**

R. We agree with the reviewer that we have used the phrase “the semi-structured approach” too often. We have carefully reviewed the Methods section and have removed some of the repetition and rewritten sentences to convey the same message. Please see the Methods section accordingly.

*C. Given our comprehensive data collection, consisting of semi-structured interviews and document analysis, we are convinced that, despite our relatively small number of interviews, we were able to present adequate variety and interpretation of our evidence.* (Page 11).

*We conducted eight semi-structured interviews which allowed us to make sure that important topics were covered, while leaving room for the participants to provide their story regarding their patient journey [30]. Given the qualitative and exploratory nature of our case study, the focus was primarily on understanding the experiences, perceptions, and attitudes of chronically ill patients during their patient journey. The semi-structured interview approach is relevant for exploratory research, as it is an effective way to gather rich data and it allows for the creation of new insights into the case under study [23].* (Page 13).

*The semi-structured approach was used to obtain insights in the touchpoints in the patient journey during different periods.* (Page 13 and 14).

**Q12. Page 13: Line 280: It may be beneficial to split the question "What is your age and gender?" into two separate questions to improve the clarity of the data collected.**

R. We have now separated the two questions to improve the clarity of the data collected.

C. *1. What is your age?* and *2. What is your gender?* (Page 14).

**Q13. Page 16: Line 289: Add references here of that literature here.**

R. We have now added the references that we used to create our coding framework.

*C. We employed this deductive, thematic analysis approach since we used a coding framework for analysis that was based on concepts and definitions derived from the literature [4, 6, 13, 19].* (Page 17).

**Q14. Page 16: Line 296: Is this text referencing style in line with PLoS One house style?**

R. We have double checked the text referencing style and can verify that the text referencing style is in accordance with the submission guidelines of PLoS ONE.

**Q15. Page 20: Line 316: Consider rephrasing "have been" to "were"**

R. We thank the reviewer for this suggestion and have changed the manuscript accordingly.

*C. To establish validity and reliability of our data, several measures were taken [33].* (Page 20).

**Q16. Page 20: Line 323: The authors mentioned that the transcripts were returned to all participants, and no comments or corrections were received. Can you kindly provide some additional information on the timeline for this process? Specifically, how long did you wait for participants to review and return the transcripts before considering it as no comments received? This information will help readers understand the study's timeline and the data verification process.**

R. We have now added a sentence in which we provide additional information on the timeline for the process of member checking.

*C. The transcripts were returned to all participants and they were given one week to review and return their transcript. A reminder was sent after one week. We received no comments and corrections, all participants agreed on their transcript.* (Page 21).

**Q17. Page 20: Line 327: Can you please clarify the decision made by the Ethics Review Board regarding the no necessity of Medical Research Involving Human Subject (WMO) approval from a Medical Ethics Committee? It would be helpful to understand the reasons behind this decision in order to ensure that ethical considerations were thoroughly evaluated and followed in the study.**

R. We apologize that we have been ambiguous about the approval of the Ethics Review Board which in turn caused confusion about the approval of the Medical Ethics Committee. We have decided to skip the sentence regarding the fact that no Medical Research Involving Human Subject approval from a Medical Ethics Committee was necessary. For this type of research, this type of approval is not required in general and as such, by including this sentence, we have been superfluous. Thus, we skipped this sentence. As a result, the text is now completely explicit about the approval of the Ethics Review Board of Catharina Hospital Eindhoven. Please see the manuscript accordingly.

*C. The Ethics Review Board of Catharina Hospital Eindhoven thoroughly evaluated and approved our study design (nWMO-2021.055). We informed the participants about the study and their rights as a participant in scientific research. All participants provided oral and written informed consent.*

**Q18. Page 20, 21: Line 334 - 341: I have reviewed the Results section and would like to recommend restructuring this section. Specifically, I believe the information about the number of patients approached, and reasons for non-participation is more appropriate in the Methods section than in the Results section. As per scientific writing conventions, the Results section should focus on presenting the study's key findings, while the Methods section provides details about the study design, recruitment process, and other pertinent information. Therefore, I would suggest revising the Results section to focus solely on the presentation of the interview results, as well as the touchpoints and digital alternatives explored in the study.**

R. We have now moved the statements related to the number of patients approached, and reasons for non-participation to the Methods section. In addition, we have moved the description of study participants to the Methods section. As such, we ensure that the Results section solely focuses on the presentation of the interview results. Please see the manuscript accordingly.

**Q19. Page 20: Line 336: In the results section, please present the interview findings in past tense since the study has already been conducted and you are reporting the results.**

R. We have now ensured that the interview findings are presented in past tense. Please see the manuscript accordingly.

**Q20. Page 21: Line 346 and 347: It is recommended to report the results using the appropriate scientific notation, such as the use of symbols and punctuation marks. For instance, instead of using a comma to separate the mean and standard deviation, it is more appropriate to use a semi-colon to separate them, as in M=50; SD=13.09. This notation is widely used in scientific writing and can help to ensure clarity and consistency in the reporting of results.**

R. We agree with the reviewer that we did not use the appropriate scientific notation to report the results. We have now ensured that we consistently apply a semi-colon to separate the mean and standard deviation.

*C. The youngest participant was 38 years old and the oldest was 78 years old (M=50.00 years; SD=13.09). On average, participants were living with their chronic illness for 18 years at the time of the interviews (M=18.00 years; SD=11.26).* (Page 12).

**Q21. Overall, these revisions will help improve the clarity and consistency of your manuscript, and ensure that readers can understand the study's methods, findings, and conclusions more easily.**

R. We agree with the reviewer that the suggested revisions have improved the clarity and consistency of our manuscript. As a result, we hope that the revised manuscript is now easier to understand for the readers of PLoS ONE.
